# Adrenal High‐Expressional CYP27A1 Mediates Bile Acid Increase and Functional Impairment in Adult Male Offspring by Prenatal Dexamethasone Exposure

**DOI:** 10.1002/advs.202413299

**Published:** 2025-02-14

**Authors:** Jiangang Cao, Wen Hu, Yawen Chen, Aihemaitijiang Ailikaiti, Ziyi Zhang, Lingbo Rong, Hong Yu, Hui Wang

**Affiliations:** ^1^ Department of Pharmacology, School of Basic Medical Sciences Wuhan University Wuhan 430071 China; ^2^ Institute of Clinical Pharmacy Research The Affiliated Nanhua Hospital Hengyang Medical School University of South China Hengyang Hunan 421001 China; ^3^ Department of Pharmacy Zhongnan Hospital of Wuhan University Wuhan 430071 China; ^4^ Hubei Provincial Key Laboratory of Developmentally Originated Disease Wuhan 430071 China

**Keywords:** adrenal steroid synthesis function impairment, bile acids, CYP27A1, prenatal dexamethasone exposure, therapeutic target

## Abstract

Prenatal dexamethasone exposure (PDE) can impact adrenal corticosteroid synthesis in adult offspring. Furthermore, the adrenal gland can autonomously synthesize bile acids, but local bile acids accumulation has cytotoxic effects. This study found that PDE increased histone 3 lysine 27 acetylation (H3K27ac) levels in the promoter region of cholesterol 27 hydroxylase (CYP27A1) and its expression, as well as total bile acids (TBA) concentrations and enhanced endoplasmic reticulum stress (ERS) and inhibit steroid synthesis in adult male offspring rat adrenal glands. However, it is reversed in females. Tracing back to the prenatal stage and in combination with cellular experiments, it is further revealed that dexamethasone can regulate glucocorticoid receptor (GR)/SET binding protein 1 (SETBP1)/CYP27A1 signal pathway, consequently cause intracellular increase of bile acids. Finally, administration of nilvadipine (CYP27A1 inhibitor) to male offspring for 4 weeks after birth resulted in the reversal of PDE‐induced adrenal morphological and functional damage. In conclusion, PDE induces fetal adrenal corticosteroid dysfunction in adult male offspring by upregulating CYP27A1 promoter region H3K27ac levels and expression in the adrenal gland through the GR/SETBP1 signaling pathway. This effect persists beyond birth, leading to bile acids local increase and subsequent enhancement of ERS, ultimately inducing cellular dysfunction in adult adrenal glands.

## Graphical abstract

Prenatal dexamethasone exposure (PDE) can induce dysfunction in adrenal steroid synthesis in adult male offspring. This study demonstrate that the mechanism is associated with the upregulation of fetal adrenal cholesterol 27 hydroxylase (CYP27A1)promoter region histone 3 lysine 27 acetylation (H3K27ac) levels and expression mediated by PDE through the glucocorticoid receptor (GR)/SET binding protein 1 (SETBP1) signaling pathway. The elevated levels of CYP27A1 H3K27ac and expression may persist postnatally, resulting in local bile acids increase in the adrenal gland, enhancedendoplasmic reticulum stress (ERS), and subsequent cellular functional impairment.

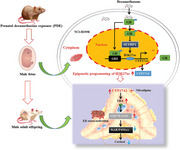

## 1 Introduction

Dexamethasone, a synthetic glucocorticoid, is extensively employed in clinical settings for the treatment of premature delivery and concomitant pregnancy complications. Its therapeutic value is manifested in its capacity to promote fetal lung maturation and mitigate the occurrence of neonatal respiratory distress syndrome, thus playing a part in reducing infant mortality rates.^[^ ^1^, ^2^, ^3^ ^]^ A survey conducted by the World Health Organization survey, which encompassed 359 institutions across 29 countries, disclosed that the prophylactic use of synthetic glucocorticoids was widespread, with an average rate of 54% and reaching up to 91% in some cases. In the Asian region specifically, ≈80% of premature infants received prophylactic treatment prior to birth. Notwithstanding the established efficacy of prenatal dexamethasone administration, an increasing number of epidemiological investigations and clinical studies drawn attention to its potential developmental toxic effects. Both human and animal experiments have demonstrated that prenatal exposure to dexamethasone can lead to low birth weight in offspring and induce developmental toxicity in multiple organs, including the bone, kidney, testes, and ovaries,^[^ ^4^, ^5^, ^6^ ^]^ ultimately resulting in heightened susceptibility to various diseases in adulthood.^[^ ^7^, ^8^, ^9^, ^10^ ^]^ The adrenal gland, as a crucial endocrine organ, primarily assumes responsibility for steroid synthesis, encompassing glucocorticoids, mineralocorticoids, and a limited quantity of sex hormones. Investigations have revealed that exposure to dexamethasone in mice during the mid to late stages of pregnancy can result in diminished adrenal steroid synthesis function of adult offspring, with a significant decrease in steroid levels observed particularly in male offspring.^[^ ^11^ ^]^ Recent studies in our department have also substantiated that prenatal dexamethasone exposure (PDE) can induce developmental toxicity in the offspring's adrenal gland,^[^ ^12^ ^]^ as well as provoke aberrant adrenal gland functionality in adulthood, characterized by inhibition in male and enhancement in female. These gender‐specific changes are associated with alterations mediated by 11‐beta‐hydroxysteroid dehydrogenase type 2 (11β‐HSD2), which is an enzyme involved in glucocorticoid action.^[^ ^13^ ^]^ However, further exploration is still required to elucidate whether there are other additional mechanisms underlying the abnormal adrenal gland function observed in PDE‐induced offspring.

Bile acids are widely acknowledged as essential physiological factors, which play a significant role in promoting nutrient absorption within the intestines and steroid secretion.^[^ ^14^, ^15^ ^]^ Epidemiological studies have shown that the serum total bile acids (TBA) level of low birth weight infants is increased after birth.^[^ ^16^ ^]^ Individuals with bile acid accumulation frequently display clinical features correlated with adrenal insufficiency,^[^ ^17^, ^18^ ^]^ Animal experiments have illustrated that increased bile acid levels in rats can lead to reduced adrenal weight and corticosterone levels,^[^ ^19^ ^]^ implying a potential induction of adrenal dysfunction due to bile acid increase. It is well‐established that cholesterol serves as the precursor for bile acid synthesis through the pathways involving cholesterol 7‐hydroxylase (CYP7A1) and cholesterol 27‐hydroxylase (CYP27A1).^[^ ^20^ ^]^ Previous investigation has indicated that CYP7A1 is expressed solely in the liver after birth.^[^ ^21^ ^]^ While CYP27A1 is expressed across multiple organs during intrauterine development and primarily exerts its function via CYP27A1 activity.^[^ ^22^ ^]^ At the cellular level, the silencing of the CYP27A1 gene in adrenal cortex cells leads to a fifty percent decrease in intracellular TBA levels.^[^ ^23^ ^]^ Given the pivotal role of CYP27A1 in maintaining cholesterol homeostasis and facilitating bile acid synthesis, the regulation of CYP27A1 has garnered considerable attention.^[^ ^24^, ^25^ ^]^ Our previous study employing non‐targeted metabolomics analysis revealed that PDE administration can augment specific bile acid components in the serum of rat offspring both prenatally and postnatally.^[^ ^26^ ^]^ This observation implies the potential involvement of the adrenal CYP27A1‐mediated bile acid synthesis pathway in regulating adrenal function in PDE progeny. However, the question of whether there is a mechanism for bile acids local increase during developmental toxicity induced by PDE in the adrenal glands remains unexplored. Moreover, it is unclear if CYP27A1 mediates this mechanism or if it stems from intrauterine factors. The above two inquiries have not been addressed thus far.

Epigenetic modification refers to heritable alterations in the regulatory mechanisms of DNA sequences without changing the DNA sequence itself, this encompasses DNA methylation, histone modification, and non‐coding RNAs such as miRNA. Mounting evidence suggests that abnormal epigenetic modifications of key genes exert a substantial influence on fetal developmental patterns and adult organ damage.^[^ ^27^, ^28^ ^]^ Our recent series of studies have also confirmed the involvement of epigenetic modifications in multi‐organ developmental programming, homeostatic changes, and susceptibility to adult‐related diseases caused by PDE.^[^ ^4^, ^5^ ^]^ Consequently, the present study aims to investigate whether PDE can induce Bile acid increase and functional damage in the adrenal glands of adult offspring by employing a stable PDE rat model. Additionally, we aim to elucidate the intrauterine epigenetic programming mechanism mediated by adrenal CYP27A1 in Bile acid increase and functional damage through animal experiments as well as human adrenal cortex cell line studies. Finally, we will explore CYP27A1 as an early intervention target and effective drug for mitigating adrenal gland functional damage in PDE adult offspring via animal and cellular intervention experiments. This study will furnish a theoretical underpinning for understanding the developmental toxicity of adrenals caused by dexamethasone application in clinical settings.

**Figure 9:**
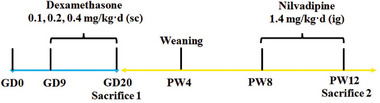
The schedule of animal treatment. From GD9‐20, pregnant Wistar rats were subcutaneously injected with dexamethasone 0.1, 0.2, and 0.4 mg kg^−1^·d. At PW8‐12, male rats were administered intragastrically with nilvadipine 1.4 mg kg^−1^·d. GD, gestational day; PW, postnatal week.

## 2 Results

### 2.1 Adult Period

#### 2.1.1 Effects of PDE on Adrenal Structure, ERS, and Steroid Synthesis in Adult Offspring Rats

**Figure 1:**
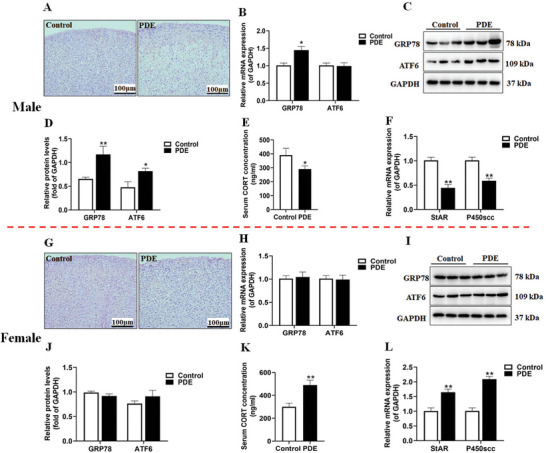
Effects of PDE on adrenal structure, ERS and steroid synthesis in male and female offspring rats at postnatal week 12. A,G) Male and female adrenal tissue H&E staining (200 ×); B,H) Male and female GRP78 and ATF6 mRNA expression; C,D,I, J) Male and female GRP78 and ATF6 protein expression; E,K) Male and female StAR and P450scc mRNA expression; F,L) Male and female serum corticosterone concentrations. Data are shown as the mean ± S.E.M., *n *= 3 for Western blot, and *n *= 8 for others. ^*^P < 0.05, ^**^P<0.01 versus control (two‐tailed *t*‐test). PDE, prenatal dexamethasone exposure; CORT, corticosterone; GRP78, glucose regulated protein 78; ATF6, activating transcription factor 6; StAR, steroidogenic acute regulatory protein; P450scc, cytochrome P450 cholesterol side chain cleavage; GAPDH, glyceraldehyde 3‐phosphate dehydrogenase.

Firstly, we examined the structural changes in the adrenal tissue of postnatal week 12 (PW12) offspring rats derived from a stable established PDE rat model [gestational day 9 to 20 (GD9‐20), subcutaneous injection of dexamethasone at a dose of 0.2 mg kg^−1^·d]. We evaluated the expression levels of endoplasmic reticulum stress (ERS)‐associated proteins, namely glucose regulated protein 78 (GRP78) and activating transcription factor 6 (ATF6), components of the steroid synthesis enzyme system, such as steroidogenic acute regulatory protein (StAR) and cytochrome P450 cholesterol side chain cleavage enzyme (P450scc), and also measured blood corticosterone levels. The adrenal tissue structure was evaluated using HE staining.^[^ ^29^, ^30^ ^]^ The results revealed that control group presented an intact adrenal cortex with a distinct structure. In contrast, male adult offspring of PDE group displayed disordered adrenal cortex structure, characterized by cell swelling, nuclear condensation, and a decreased cell count (**Figure** **1**A). Real‐time quantitative reverse transcription‐polymerase chain reaction (RT‐qPCR) and immunohistochemistry assays revealed that the mRNA and protein expression levels of GRP78 in the adrenal glands of male adult offspring from the PDE group were higher compared to those of the controls (Figure 1B–D). Additionally, the protein expression level of ATF6 was elevated in these male adult offspring (Figure 1C,D), although the mRNA expression remained unchanged. Furthermore, we found that compared to the control group, the mRNA expression levels of StAR and P450scc, as well as blood corticosterone levels were decreased in the adrenal glands of PDE male adult offspring (Figure 1E,F).

Subsequently, we turned our attention to observe the fluctuations in the indicators in female offspring. Intriguingly, when juxtaposed with the control, no pronounced or statistically significant changes were discernible, neither in the anatomical architecture of the adrenal glands (Figure 1G) nor in the mRNA and protein expression profiles of GRP78 and ATF6 nestled in the adrenal tissue of PDE female adult offspring (Figure 1H–J). On the contrary, a rather conspicuous elevation was noted in the mRNA expression tiers of StAR and P450scc, concomitantly accompanied by a hike in the circulating levels of blood corticosterone in the adrenal glands of PDE female adult offspring rats (Figure 1K,L). In summary, PDE can induce adrenal ERS, structure and steroid synthesis changes in adult offspring, and there are gender differences.

#### 2.1.2 Effects of PDE on Adrenal Bile Acid Synthesis, Transport Function, and TBA Levels in Adult Offspring Rats

**Figure 2:**
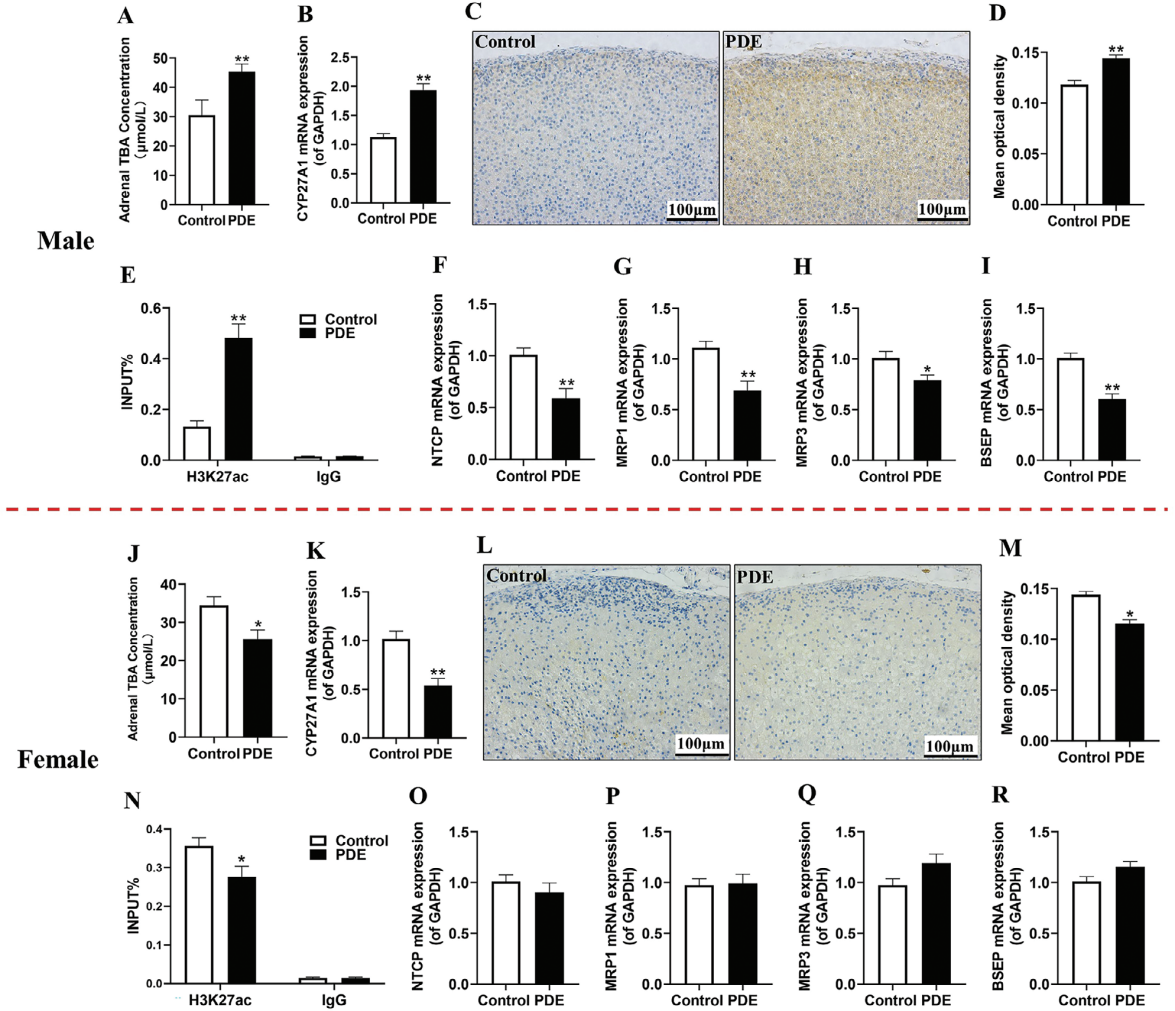
Effects of PDE on adrenal bile acid synthesis, transportation function and TBA levels in male and female offspring rats at postnatal week 12. A,J) Male and female TBA concentrations; B,K) Male and female CYP27A1 mRNA expression; C,L) Male and female CYP27A1 IHC staining (200 ×); D,M) Male and female CYP27A1 mean optical density; E,N) Male and female CYP27A1 H3K27ac levels; F–I,O‐R) Male and female NTCP, MRP1/3 and BSEP mRNA expression. Data are shown as the mean ± S.E.M., *n *= 3 for ChIP assay, *n *= 5 for IHC staining (sections of each group were selected and five random fields of each section scored) and *n *= 8 for other experiments. ^*^P < 0.05, ^**^P < 0.01 versus control (two‐tailed *t*‐test). PDE, prenatal dexamethasone exposure; TBA, total bile acids; CYP27A1, cytochrome P450 family 27 subfamily A member 1; NTCP, sodium taurocholate cotransporting polypeptide; MRP1/3, multidrug resistance‐associated protein1/3; BSEP, bile salt export pump; H3K27ac, histone 3 lysine 27 acetylation; IgG, immunoglobulin G; GAPDH, glyceraldehyde 3‐phosphate dehydrogenase; IHC, immunohistochemical.

In an endeavor to unravel the underlying mechanism responsible for the inhibition of adrenal steroid synthesis in PDE adult offspring rats, we meticulously scrutinized the alterations occurring in adrenal bile acid synthesis, the expression patterns of transport‐related genes, as well as the levels of TBA. Initially, our research findings disclosed that, in contrast to the control group, the local TBA levels in the adrenal glands of PDE male adult offspring rats exhibited a remarkably significant elevation (**Figure** **2**A). It is widely acknowledged that bile acid synthesis is predominantly regulated by two principal pathways: namely, the “classical pathway,” which is under the aegis of CYP7A1, and the “alternative pathway,” overseen by CYP27A1.^[^ ^31^ ^]^ Moreover, we undertook a comprehensive screening and in‐depth analysis of the changes in the expression of enzymes implicated in bile acid synthesis (Figure S1A, Supporting Information). It was observed that, in consonance with previous reports,^[^ ^21^ ^]^ CYP7A1 remained conspicuously absent in the adrenal glands of adult offspring rats. Significantly, when compared to the control counterparts, the mRNA and protein levels of CYP27A1 in the adrenal glands of PDE male adult offspring rats were found to be substantially augmented (Figure 2B–D). As is well‐established in the scientific realm, histone acetylation possesses the capacity to instigate chromatin relaxation and consequently bolster gene expression.^[^ ^32^ ^]^ In this regard, we conducted a concurrent screening of multiple histone acetylation levels in the promoter region of CYP27A1. The outcomes unequivocally demonstrated that PDE led to a pronounced upsurge in the histone 3 lysine 27 acetylation (H3K27ac) levels in the adrenal glands of male adult offspring (Figure 2E). However, it is noteworthy that the changes in the H3K9ac and H3K14ac levels bore no correlation with the expression of CYP27A1 in male subjects (Figure S1B, Supporting Information). Moving forward, we directed our attention toward evaluating the expression of bile acid transporters, encompassing both uptake and efflux transporters. Our investigations unearthed that the mRNA expression of the bile acid uptake transporter sodium taurocholate co‐transporting polypeptide (NTCP), along with that of the efflux transporters cholesterol efflux gene multidrug resistance‐associated protein 1 (MRP1), MRP3 and bile salt export pump (BSEP), registered a significant decline in the adrenal glands of PDE male adult offspring rats (Figure 2F–I).

Subsequently, we shifted to observing the variations in the indicators in the female offspring cohort. Upon comparison with the control group, it was discerned that the local TBA levels in the adrenal glands of PDE female adult offspring rats manifested a significant decline (Figure 2J). Additionally, we meticulously screened the alterations in the expression of bile acid synthesis enzymes (Figure S1C, Supporting Information) as well as the multiple histone acetylation levels in the promoter region of CYP27A1 (Figure S1D, Supporting Information). The results revealed that, in contrast to the control specimens, both the mRNA and protein levels of CYP27A1, along with the H3K27ac levels, were noticeably reduced in the adrenal glands of PDE female adult offspring rats (Figure 2K–N). Subsequently, further observation indicated that there were no conspicuous alterations in the mRNA expression of NTCP, MRP1/3, and BSEP in the adrenal glands of the female offspring rats (Figure 2O–R). In summation, it becomes evident that PDE predominantly governs adrenal bile acid synthesis through its modulation of CYP27A1, with pronounced gender‐based discrepancies being observable.

#### 2.1.3 Effects of CDCA on ERS and Steroid Synthesis in Human Adrenal Cortex Cells

**Figure 3:**
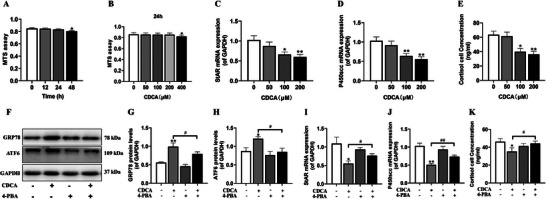
Endoplasmic reticulum stress‐mediated cortisol synthesis function changes in human fetal adrenal cortical cells induced by CDCA. A,B) Cell viability in different time and concentrations; C,D) StAR and P450scc mRNA expression in different concentrations; E: The cell supernatant cortisol level in different concentrations; F–H) GRP78 and ATF6 protein expression in presence of 4‐PBA; I,J) StAR and P450scc mRNA expression in presence of 4‐PBA; K) The cell supernatant cortisol level in presence of 4‐PBA. Data are shown as the mean ± S.E.M., *n *= 3 for Western blot and *n *= 6 for other experiments. ^*^P < 0.05, ^**^P < 0.01 versus control; *^#^P *< 0.05, *^##^P *< 0.01 versus CDCA group (two‐tailed *t*‐test or one‐way ANOVA). CDCA, chenodexycholic acid; StAR, steroidogenic acute regulatory protein; P450scc, cytochrome P450 cholesterol side chain cleavage; GRP78, glucose regulated protein 78; ATF6, activating transcription factor 6; 4‐PBA, 4‐phenylbutyric acid; GAPDH, glyceraldehyde 3‐phosphate dehydrogenase.

Through correlation analysis, we found that the concentration of TBA in the adrenal glands of adult male offspring of PDE was positively correlated with the expression of endoplasmic reticulum stress markers (GRP78 and ATF6) and the content of endogenous corticosterone (Figure S2A–C, Supporting Information). To validate the augmentative influence of elevated bile acids on local ERS in the adrenal gland, as well as its suppressive effect on cortisol synthesis, we subjected NCI‐H295R cells to treatment with diverse concentrations of chenodeoxycholic acid (CDCA),^[^ ^33^ ^]^ or co‐treated them in tandem with an ERS inhibitor, 4‐phenylbutyric acid (4‐PBA).^[^ ^34^ ^]^ Subsequently, we meticulously monitored the alterations in ERS markers, steroid synthetase expression, and cell supernatant cortisol levels. The MTS assay outcomes signified that exposure to 200 µm CDCA for varying time intervals induced cytotoxicity at the 48‐h mark (**Figure** **3**A). Likewise, when the cells were treated with an array of CDCA concentrations for 24 h, cytotoxicity emerged at a concentration of 400 µm (Figure 3B). Moreover, we ascertained that CDCA elicited a dose‐dependent reduction in the mRNA expression of steroid synthetases, namely StAR and P450scc, as well as a decline in cortisol levels in the cell supernatant (Figure 3C–E). Consequently, we opted to employ a cell model that had been treated with 200 µm CDCA for 24 h to execute subsequent intervention experiments. The results divulged that, in comparison to the control cohort, those cells treated with CDCA manifested an elevated expression of ERS markers, GRP78 and ATF6. However, this upsurge was counteracted by co‐treatment with 5 mm 4‐PBA (Figure 3F–H). Concurrently, 4‐PBA efficaciously reversed the CDCA‐induced diminution in the mRNA expression of adrenal StAR and P450scc, as well as the cortisol levels in the cell supernatant (Figure 3I–K). These revelations imply that the activation of ERS by CDCA hampers the glucocorticoid (cortisol) synthesis function of adrenal cortex cells.

### 2.2 Intrauterine Period

#### 2.2.1 Effect of PDE on CYP27A1 Expression Related Bile Acid Synthesis and Its Epigenetic Mechanism in Male Fetal Rats

**Figure 4:**
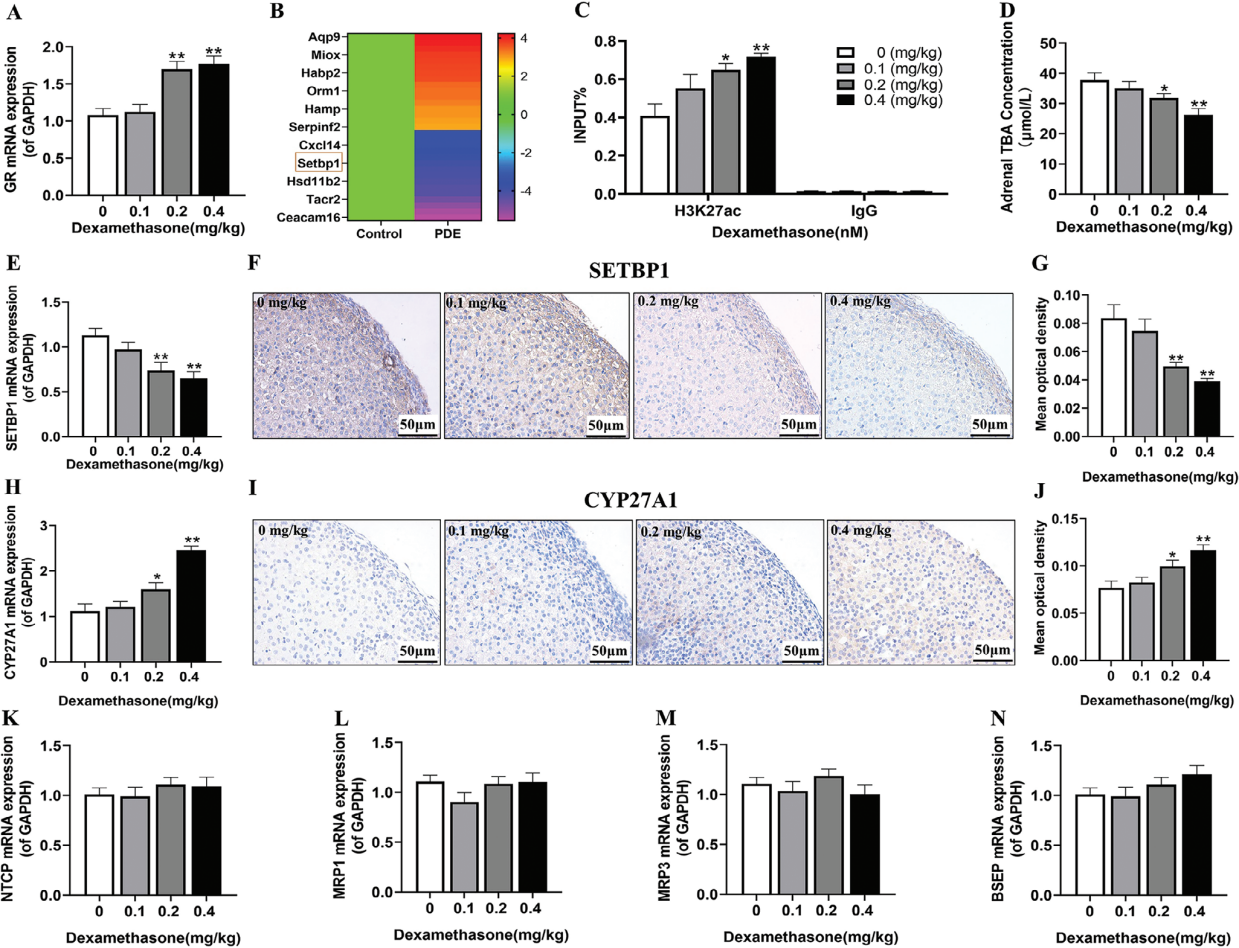
Effects of PDE on adrenal CYP27A1 expression‐related bile acid synthesis and epigenetic mechanism in male fetal rats at gestational day 20. A) GR mRNA expression; B) Heat map of differentially expressed genes; C) CYP27A1 H3K27ac level; D) adrenal TBA level; E) SETBP1 mRNA expression; F) SETBP1 protein expression by IHC staining (400 ×); G) SETBP1 mean optical density; H) CYP27A1 mRNA expression; I) CYP27A1 protein expression by IHC staining (400 ×); J) CYP27A1 mean optical density; K) NTCP mRNA expression; L,M) MRP1/3 mRNA expression; N) BSEP mRNA expression. Data are shown as the mean ± S.E.M., six pairs of fetal adrenals from two littermates were pooled for homogenization into one sample; *n *= 5 for IHC staining (sections of each group were selected and five random fields of each section scored), *n *= 3 for ChIP assay and *n *= 8 for others. ^*^P < 0.05, ^**^P < 0.01 versus control (two‐tailed *t*‐test). PDE, prenatal dexamethasone exposure; CYP27A1, cholesterol 27‐hydroxylase; GR, glucocorticoid receptor; SETBP1, SET binding protein1; TBA, total bile acids; H3K27ac, histone 3 lysine 27 acetylation; NTCP, sodium taurocholate cotransporting polypeptide; MRP1/3 cholesterol efflux gene multidrug resistance‐associated protein1/3; BSEP, bile salt export pump; IgG, immunoglobulin GAPDH, glyceraldehyde 3‐phosphate dehydrogenase; IHC, immunohistochemical.

To uncover the fundamental mechanism responsible for the elevated expression and increased H3K27ac levels of CYP27A1 in PDE adult male offspring rats, we investigated the expression, H3K27ac level, and associated signaling of CYP27A1 in fetal adrenal glands. Dexamethasone was subcutaneously injected at doses of 0.1, 0.2, and 0.4 mg kg^−1^·d from GD9‐20. Initially, we examined the alterations in expression levels of bile acid synthesis enzymes in male fetal rats. Interestingly, our findings revealed that CYP7A1, which is typically associated with liver,^[^ ^35^ ^]^ can also be expressed in the fetal adrenal glands. However, when compared to the control group, the mRNA expression of CYP7A1 in the adrenal glands of PDE male fetal rats was significantly decreased, while CYP27A1 increased. (Figure S3A, Supporting Information). p   In addition, an exploration of the glucocorticoid receptor (GR) expression within the adrenal gland disclosed an upregulation of GR mRNA levels in the PDE group (**Figure** **4**A). Considering the pivotal role that epigenetic enzymes play in regulating gene expression levels, we employed gene chips and RT‐qPCR to evaluate the expressions of several epigenetic enzymes. Our findings showed that the mRNA expression of SET binding protein 1 (SETBP1) was markedly reduced in the PDE group when compared with the control group (Figure 4B). Meanwhile, RT‐qPCR results showed SIRT1 expression exhibited an increase, whereas the expressions of SIRT2‐6 remained unaltered (Figure S3B, Supporting Information). Subsequently, we assessed the acetylation status of multiple histones in the promoter region of the fetal rat CYP27A1 gene. Notably, a significant elevation was specifically detected in the H3K27ac level in the adrenal glands of PDE male fetal rats (Figure 4C). In contrast, the H3K9ac and H3K14ac levels did not show any significant changes (Figure S3C, Supporting Information). Moreover, a dose‐dependent decrease in TBA levels was observed in the adrenal glands of PDE male fetal rats (Figure 4D). RT‐qPCR and immunohistochemistry further verified a substantial reduction in both the mRNA and protein levels of SETBP1 in the PDE group (Figure 4E‐G).Our research further demonstrated a clear dose‐dependent increase in both the mRNA and protein expression levels of CYP27A1 in the adrenal glands of PDE fetal rats (Figure 4H‐J). Subsequently, further observation indicated that there were no remarkable alterations in the mRNA expression of NTCP, MRP1/3, and BSEP in the adrenal glands of the male fetal rats (Figure 4K‐N). In summary, these results suggest that PDE increases the expression of CYP27A1 by upregulating the H3K27ac level in the promoter region of adrenal CYP27A1, but inhibits the expression of CYP7A1, ultimately leading to a decrease in the adrenal TBA level in male fetal rats.

Simultaneously, alterations in the indicators were also observed in the adrenal glands of female fetal rats. Like PDE male fetal rats, a dose‐dependent increase was detected in both the H3K27ac levels and expression of CYP27A1(Figure S4A–D, Supporting Information). And the mRNA expression of CYP7A1 decreased (Figure S3D, Supporting Information) accompanied by a reduction in TBA levels (Figure S4E, Supporting Information). Additionally, the mRNA expression of the GR increased significantly (Figure S4F, Supporting Information). When we screened epigenetic enzymes using chip technology, we found no difference in SETBP1 expression in the adrenal glands of PDE female fetal rats (Figure S4G, Supporting Information). However, the results from RT‐qPCR and immunohistochemistry showed a different pattern compared to their male counterparts. In PDE female fetal rats, the mRNA and protein expression of SETBP1 exhibited a dose‐dependent increase (Figure S4H–J, Supporting Information).

#### 2.2.2 Dexamethasone Directly Regulates the Transcriptional Expression of CYP27A1 in Human Adrenal Cortex Cells through GR

**Figure 5:**
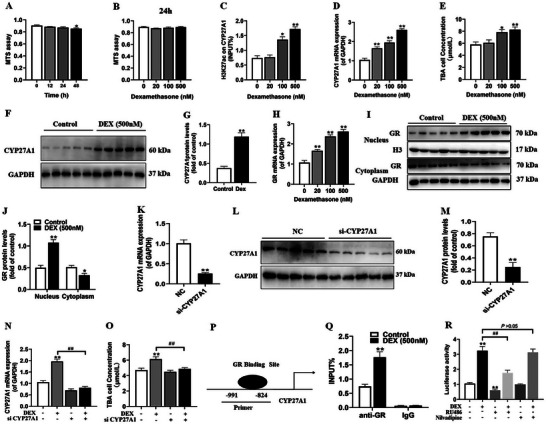
Effects of DEX on CYP27A1 expression‐related bile acid synthesis in human adrenal cortical cells. A,B) Cell viability in different time and concentrations; C) CYP27A1 H3K27ac levels in different concentrations; D) CYP27A1 mRNA expression in different concentrations; E) TBA levels in different concentrations; F,G) CYP27A1 protein expression; H) GR mRNA expression in different concentrations; I,J) GR protein expression in cytoplasm and nucleus after DEX treatment; K–M) CYP27A1 mRNA and protein expression after CYP27A1‐siRNA transfection; N) CYP27A1 mRNA expression in presence of CYP27A1‐siRNA and DEX; O) TBA levels in presence of CYP27A1‐siRNA and DEX; P) The predicted promoter region of GR enrichment in CYP27A1; Q) GR enrichment in CYP27A1 promoter region after DEX treatment; R) The relative luciferase activities after treated with DEX and/or RU486, nivaldipine. Data are shown as the mean ± S.E.M., *n *= 5 for protein expression, *n *= 3 for ChIP assay, and *n *= 6 for others. ^*^P < 0.05, ^**^P < 0.01 versus control; *^##^P *< 0.01 versus DEX group (two‐tailed *t*‐test or one‐way ANOVA). DEX, dexamethasone; CYP27A1, cholesterol 27‐hydroxylase; H3K27ac, histone 3 lysine 27 acetylation; TBA, total bile acids; IgG, immunoglobulin G; GR, glucocorticoid receptor; RU486, mifepristone; GAPDH, glyceraldehyde 3‐phosphate dehydrogenase.

As above mentioned, we observed that PDE could elevate the levels of H3K27ac modification on CYP27A1 and enhance its expression in the fetal rat adrenal gland. To corroborate this phenomenon at the cellular level, we subjected human adrenal cortex cells (NCI‐295R cells) to treatments with a range of dexamethasone concentrations, spanning from 0 to 500 nm. The MTS results showed that treating the cells with 500 nm dexamethasone for 48 h significantly reduced cell viability (**Figure** **5**A). Meanwhile, treatment with dexamethasone concentrations in the 0–500 nm range for 24 h had no appreciable effect on cell viability (Figure 5B). Based on these outcomes, we established that a 24 h treatment with 500 nm dexamethasone was the most suitable experimental condition. Our subsequent findings indicated that, in comparison to the control group, dexamethasone treatment induced a concentration‐dependent increase in H3K27ac levels of CYP27A1, its mRNA expression, and TBA in the cell supernatant (Figure 5C–E). Moreover, when compared to controls, dexamethasone treatment promoted the expression of CYP27A1 protein (Figure 5F,G). In addition, we observed that dexamethasone treatment upregulated the mRNA expression of the GR (Figure 5H). It also led to an increase in the nuclear localization of GR protein, accompanied by a decrease in the cytoplasmic expression levels of the GR protein (Figure 5I,J). These findings suggest that dexamethasone facilitates the nuclear translocation of GR.

To validate the regulatory function of GR in TBA production through CYP27A1, si‐CYP27A1 was transfected into NCI‐H295R cells followed by a 24 h treatment with 500 nm dexamethasone. The results clearly showed that after transfecting the si‐CYP27A1 plasmid, both the mRNA and protein expression levels of CYP27A1 significantly decreased (Figure 5K–M). This transfection also reversed the elevation of CYP27A1 mRNA expression and TBA levels induced by dexamethasone (Figure 5N,O). Subsequently, through bioinformatics analysis, we predicted the existence of GR binding sites in the promoter region (‐991—‐824) of the CYP27A1 gene (Figure 5P). Further verification using ChIP‐qPCR technology revealed that dexamethasone promoted binding of GR to the CYP27A1 promoter region (Figure 5Q). Finally, to confirm whether dexamethasone could enhance the transcriptional expression of CYP27A1 through GR activation, we performed a transient transfection of the human CYP27A1 promoter luciferase reporter gene plasmid along with the Renilla luciferase plasmid into NCI–H295R cells. The cells were treated with dexamethasone and/or RU486 (a GR inhibitor), as well as nilvadipine (a CYP27A1 inhibitor^[^ ^36^ ^]^) for 24 h before measuring the ratio of firefly luciferase to Renilla luciferase. The results indicated that treatment with dexamethasone alone led to a significant increase in CYP27A1 transcriptional activity. However, this effect was reversed by the administration of RU486 (Figure 5R). Notably, a 5 µm concentration of nilvadipine did not impact the dexamethasone‐induced increase in CYP27A1 transcriptional activity (Figure 5R). Collectively, these findings suggest that dexamethasone promotes CYP27A1 transcriptional activity by activating GR and enhancing its binding affinity to the promoter region.

#### 2.2.3 GR/SETBP1/CYP27A1 Acetylation‐Mediated Dexamethasone‐Induced High Expression of CYP27A1 in Human Adrenal Cortex Cells

**Figure 6:**
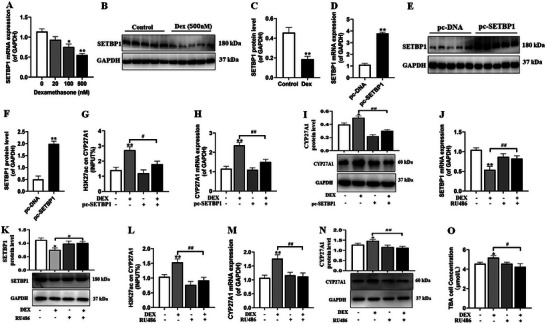
Effects of DEX on CYP27A1 expression‐related epigenetic mechanism in human adrenal cortical cells. A–C) The SETBP1 mRNA and protein expression after dexamethasone treatment; D–F) The SETBP1 mRNA and protein expression after pcDNA‐SETBP1 transfection; G) CYP27A1 H3K27ac level in the presence of SETBP1 overexpression; H,I) CYP27A1 mRNA and protein expression in the presence of SETBP1 overexpression and DEX; J,K) SETBP1 mRNA and protein expression in presence of RU486 treatment and DEX; L) CYP27A1 H3K27ac level in the presence of RU486 treatment and DEX; M,N) CYP27A1 mRNA and protein expression in the presence of RU486 treatment and DEX; O) Cell supernatant TBA concentration in the presence of RU486 treatment and DEX. Data are shown as the mean ± S.E.M., *n *= 5 for Western blot, *n *= 3 for ChIP assay and *n *= 6 for others. ^*^P < 0.05, ^**^P < 0.01 versus control or NC group or pc‐NDA group; *^#^P *< 0.05, *^##^P *< 0.01 versus DEX group (two‐tailed *t*‐test or one‐way ANOVA). DEX, dexamethasone; CYP27A1, cholesterol 27‐hydroxylase; TBA, total bile acids; SETBP1, SET binding protein 1; H3K27ac, histone 3 lysine 27 acetylation; IgG, immunoglobulin G; GR, glucocorticoid receptor; RU486, mifepristone; GAPDH, glyceraldehyde 3‐phosphate dehydrogenase.

At the cellular level, we delved deeper into the epigenetic regulatory mechanism underlying the enhanced expression of CYP27A1 induced by dexamethasone. Firstly, our findings clearly indicated that dexamethasone exerted a significant suppressive effect on both the mRNA and protein expression of SETBP1 (**Figure** **6**A–C). Subsequently, we transfected NCI‐H295R cells with 2.5 µg of the pc‐SETBP1 overexpression plasmid and then treated them with 500 nm dexamethasone for 24 h. Transfection with the pc‐SETBP1 plasmid led to a substantial increase in both the mRNA and protein expression of SETBP1 (Figure 6D–F). More importantly, this increase in SETBP1 expression subsequently reversed the elevation of CYP27A1 H3K27ac levels, as well as the enhanced mRNA and protein expression of CYP27A1 induced by dexamethasone treatment (Figure 6G–I). Finally, we co‐treated NCI‐H295R cells with 500 nm dexamethasone and 2.5 µm of the GR inhibitor RU486 for 24 h. RU486 effectively counteracted the inhibitory effect of dexamethasone on SETBP1 mRNA and protein expression (Figure 6J,K). Concomitantly, it attenuated the increased in CYP27A1 H3K27ac levels, as well as the upregulated mRNA and protein expression of CYP27A1 and the TBA levels induced by dexamethasone treatment (Figure 6L–O). In summary, dexamethasone can activate GR to suppress SETBP1 gene transcription, resulting in an elevation in H3K27ac modification on the CYP271 gene promoter region and ultimately leading to upregulated expressions of CYP27A1 gene transcription and TBA level in human adrenal cortex cells.

### 2.3 Postnatal Intervention: Reversal Effect of CYP27A1 Inhibitor Nilvadipine on Adrenal Cortex Cell Dysfunction of PDE Male Offspring Rats

**Figure 7:**
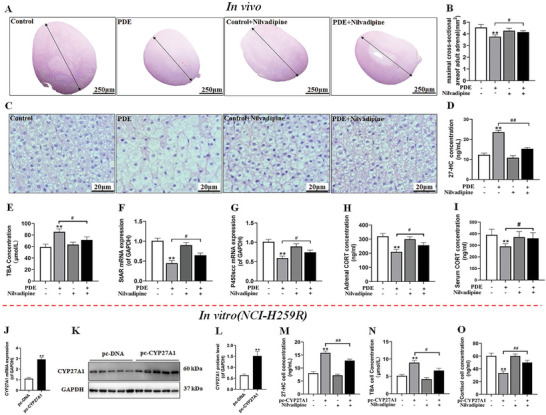
Effects of nilvadipine on adrenal dysfunction in PDE male adult offspring rats and CYP27A1‐overexpression NCI‐H295R cells. A,C) Adrenal tissue H&E staining (80 ×, 400 ×); B) Adrenal entire cross sectional area (*n *= 5); D) Adrenal 27‐HC content; E) Adrenal TBA content; F,G) Adrenal StAR and P450scc mRNA expression; H) Adrenal corticosterone content; I) Serum corticosterone concentration; J) CYP27A1 mRNA expression; K,L) CYP27A1 protein expression; M) 27‐HC concentration; N) TBA concentration; O) Cortisol concentration; J‐O were detected in NCI‐H295R cell after treated with pcDNA‐CYP27A1 or/and nilvadipine. Data are shown as the mean ± S.E.M., *n *= 5 for protein expression, and *n *= 6 for others. ^*^P < 0.05, ^**^P < 0.01 versus control or pc‐NDA group; *^#^P *< 0.05, *^##^P *< 0.01 versus PDE group or pc‐CYP27A1 group (two‐tailed *t*‐test or one‐way ANOVA). CYP27A1, cholesterol 27‐hydroxylase; PDE, prenatal dexamethasone exposure; 27‐HC, 27‐hydroxyl cholesterol; TBA, total bile acids; StAR, steroidogenic acute regulatory protein; P450scc, cytochrome P450 cholesterol side chain cleavage; CORT, corticosterone; GAPDH, glyceraldehyde 3‐phosphate dehydrogenase.

The study revealed that nilvadipine is an effectively inhibitor of CYP27A1.^[^ ^36^ ^]^ To investigate whether CYP27A1 could serve as an early preventive target for adrenal dysfunction in male offspring after birth, we initially an investigation. First, nilvadipine was administered at a dose of 1.4 mg kg^−1^·d via gavage for a period of 4 weeks at an early postnatal stage (PW8). Subsequently, we evaluated the therapeutic effect of nilvadipine on adrenal steroid‐synthesis dysfunction in adult male offspring from the PDE group. Our results showed that, compared to the control group, the PDE group had a significant reduction in the maximum cross sectional area of the adrenal glands (**Figure** **7**A,B). The adrenal gland cell structure in the PDE group was disrupted, characterized by cell swelling, nuclear condensation, and a decreased cell count. However, treatment with nilvadipine significantly ameliorated these structural damages observed in the adrenal glands of adult male offspring from the PDE group (Figure 7C). Furthermore, our findings confirmed that nilvadipine reversed elevated levels of local 27‐hydroxycholesterol (27‐HC) and TBA in the adrenal glands caused by PDE (Figure 7D,E). It also suppressed mRNA expression of steroid synthesis enzymes, such as StAR and P450scc (Figure 7F,G). Additionally, nilvadipine restored the endogenous corticosterone content in the adrenal glands (Figure 7H) and increased blood corticosterone levels (Figure 7I). In summary, the nilvadipine effectively improves structural damage to adrenal tissue and corticosterone synthesis dysfunction in PDE adult male offspring.

Simultaneously, we delved into the impact of nilvadipine on dexamethasone‐induced dysfunction in adrenal cortical cells at a cellular level. Initially, to mimic the high CYP27A1 expression observed in male offspring after birth, NCI‐H295R cells were transfected with a pc‐CYP27A1 overexpression plasmid. Twelve hour post‐transfection, the cells were treated with 5 µm nilvadipine for 24 h. The results clearly showed that pc‐CYP27A1 overexpression plasmid significantly upregulated both mRNA and protein expression of CYP27A1 (Figure 7J–L). In contrast, nilvadipine effectively counteracted the elevation of 27‐HC and TBA levels. Moreover, it reduced cortisol levels induced by CYP27A1 overexpression (Figure 7M–O). In summary, our findings suggest that inhibition of CYP27A1 by nilvadipine can ameliorate adrenal cortical cell dysfunction caused by CYP27A1 overexpression.

## 3 Discussion

### 3.1 The Basis of Drug Use in PDE Rats and PDE‐Induced Impairment of Adult Offspring's Adrenal Steroidogenesis

Clinically, synthetic glucocorticoids, such as dexamethasone or betamethasone, are routinely administered to pregnant women with a predisposition for preterm birth between 23–34 weeks of gestation. This treatment aims to promote fetal lung tissue maturation and prevent respiratory distress syndrome in premature infants after delivery. The National Institutes of Health recommends intramuscular administration of prenatal dexamethasone therapy at a dosage of 6 mg every 12 h, with a total of 4 doses per treatment course.^[^ ^37^ ^]^ For an adult woman weighing ≈60 kg, this regimen is equivalent to 0.2 mg kg^−1^·d. Considering the dose conversion ratio between humans and rats, which is based on a 1:6.17 body surface area),^[^ ^38^ ^]^ the clinical dosage for pregnant women corresponds to ≈1.234 mg kg^−1^·d in rats. Considering this, the dexamethasone doses used in this study (0.1, 0.2, and 0.4 mg kg^−1^·d) are considered appropriate for simulating mid‐to‐late pregnancy treatments in a pre‐clinical context. These doses were administered via subcutaneous injection to pregnant Wistar rats to establish a rat model mimicking human prenatal exposure. Previously, we measured a serum dexamethasone concentration of 230 ± 61 nm in fetal rats in the 0.2 mg kg^−1^·d model.^[^ ^6^ ^]^ Based on these findings, for our cell experiment, we selected dexamethasone concentrations of 0, 20, 100, and 500 nm. The 500 nm concentration, which closely approximates fetal blood levels, was chosen for further mechanistic investigations.

Adrenal function injury is defined as the suppression of adrenal function caused by prolonged exposure to exogenous factors, particularly glucocorticoids. This condition is typically manifested by a decline in the expression of steroid synthases and a reduction cortisol (ketone) levels.^[^ ^39^ ^]^ Prior animal experiments have shown that exposing mice to dexamethasone during mid‐to‐late pregnancy significantly diminishes the expression of adrenal steroid synthetase StAR in adult male offspring, consequently leading to decreased steroid levels.^[^ ^40^ ^]^ Our previous research has also revealed that PDE inhibits the expression of 11β‐HSD2 in adult male adrenal glands, thereby resulting in reduced adrenal steroid synthesis.^[^ ^13^ ^]^ In the currents study, we observed an increase in local adrenal bile acid levels, a decrease in the expression of steroid synthases expression, and a reduction in blood corticosterone levels in PDE male offspring rats. In contrast, PDE female offspring rats exhibited opposing outcomes. These findings strongly suggest that PDE can compromise the adrenal steroid synthesis function in adult male rats. Moreover, they provide direct evidence of the developmental toxicity associated with the clinical use of dexamethasone and the susceptibility of offspring to adult‐related diseases. This research underscores the importance of carefully considering the potential long‐term consequences of prenatal glucocorticoid exposure in clinical practice, as it may have far‐reaching implications for the health of the offspring in adulthood.

### 3.2 CYP27A1 Overexpression/Bile Acid Increase Mediates Enhanced ERS and Functional Damage in PDE Adult Male Offspring Adrenal Glands

Studies have demonstrated that bile acids possess cytotoxic properties, especially toward metabolically active cells. They achieve this by disrupting mitochondrial function and interrupting the electron transport chain. As a consequence, ATP production is impaired, leading to cellular energy depletion.^[^ ^41^, ^42^ ^]^ Clinical investigations have reported that elevated bile acids concentrations can inhibit the synthesis of adrenal corticosteroid.^[^ ^43^ ^]^ Findings from animal models have indicated that in rats, increased bile acid levels can lead to a reduction in adrenal weight and corticosterone levels,^[^ ^19^ ^]^ strongly suggesting an inhibitory effect on adrenal corticosteroid synthesis. In our present study, we also observed that the local TBA levels in the adrenal glands of PDE adult male rats were significantly higher (45.4 ± 2.6 µm) compared to those in the control group (30.5 ± 5.2 µm). When considering the drug – dosage conversion rate between rats and humans, the estimated corresponding concentration of bile acid in the human adrenal gland is ≈280.1 ± 15.9 µm.

Therefore, considering CDCA as the secondary bile acid synthesized via CYP27A1 and integrating with the experimental bile acid concentrations (100–400 µm) reported in the literature,^[^ ^33^ ^]^ we simulated a high bile acid environment in the adrenal glands of adult male rats at the cellular level. We used super‐physiological (yet non‐pathological) concentrations of CDCA, specifically 50, 100, 200, and 400 µm. Our experiments confirmed that CDCA can inhibit cortisol production in adrenal cortical cells. This finding implies that elevated CDCA levels contribute to the impairment of corticosteroid synthesis in adrenal glands of PDE adult male rat. However, the exact mechanism by which high bile acids, induced by PDE, inhibit adrenal corticosteroid synthesis remains unclear. Some studies suggests that bile acids may induce oxidative damage by stimulating the production of oxygen free radicals in mitochondria.^[^ ^44^, ^45^, ^46^ ^]^ ERS, a common form of oxidative stress, is known to trigger apoptosis in adrenal cells.^[^ ^47^, ^48^ ^]^ In animal studies, drug‐induced oxidative stress has been demonstrated to inhibit adrenal corticosteroid synthesis and consequently reduce corticosterone levels in rats.^[^ ^30^ ^]^ In our study, we observed elevated expression levels of ERS markers, GRP78 and ATF6, in PDE adult male rat adrenal glands, indicating the activation of ERS. Subsequent cell‐based experiments further confirmed that treatment with a high concentration of CDCA (200 µm) increased the protein expression levels of GRP78 and ATF6 in NCI – H295R cells. Notably, co‐treatment with the ERS inhibitor 4‐PBA reversed the effects induced by CDCA and restored cortisol levels. In summary, our findings suggest that the increase of bile acids mediates activation of adrenal ERS induced by PDE during adulthood. This activation ultimately leads to the inhibition of steroid synthesis.

Bile acid synthesis is known to occur through two distinct pathways. The classic pathway, also referred to as the neutral pathway and mediated by CYP7A1, is responsible for ≈90% of bile acid synthesis. In contrast, the alternative pathway, or the acidic pathway, which is mediated by CYP27A1, contributes ≈10% of the TBA production.^[^ ^49^ ^]^ The alternative pathway involves a series of enzymatic reactions that take place in multiple cellular compartments, including the endoplasmic reticulum, mitochondria, cytoplasm, and peroxisomes.^[^ ^50^ ^]^ We comprehensively evaluate the expression changes of key enzymes involved in bile acid synthesis in the adrenal tissues of PDE male offspring rats, both before and after birth. Notably, The level of CYP7A1 in PDE male fetal rats is decreased, which potentially be one of the reasons for the decrease in local TBA levels in the adrenal glands of PDE fetal rats. Moreover, it's important to note that adult adrenal glands do not express CYP7A1,^[^ ^21^ ^]^ but instead express CYP27A1. Knocking down the CYP27A1 gene leads to a 50% reduction in TBA levels in adrenal cortical cells.^[^ ^23^ ^]^ These findings suggest that the adrenal glands have the autonomous ability to synthesize bile acids via the CYP27A1 pathway. In this study, we observed an increase in CYP27A1 expression in the adrenal glands of PDE male offspring both prenatally and postnatally. At the cellular level, treatment with dexamethasone led to the upregulation of CYP27A1 expression and an increase in TBA levels in NCI‐H295R cells. Conversely, when CYP27A1 was knocked down using siRNA, the dexamethasone‐induced elevation of CYP27A1 expression and TBA levels was reversed. Collectively, these findings indicate that the sustained high expression of CYP27A1 contributes to the elevated local bile acid concentrations in the adrenal glands of PDE adult male offspring.

### 3.3 Dexamethasone Increases the Adrenal CYP27A1 H3K27ac Levels and Expression by GR/ SETBP1 (Dual Pathway)

Glucocorticoids, such as dexamethasone, primarily exert their effects through the ligand‐dependent nuclear transcription factor, the GR. Once bound to glucocorticoids, GR gets activated and then modulates gene expression. It not only directly binds to the promoters of target genes but also triggers epigenetic modifications and expression changes through other signaling molecules, thereby inducing alterations in fetal tissue structure. These persistent changes have been confirmed in previous studies.^[^ ^51^, ^52^ ^]^ Previous studies have demonstrated that dexamethasone can activate GR to induce CYP27A1 expression and activity.^[^ ^53^ ^]^ In this study, we also predicted the presence of GRE binding sites in the promoter region of CYP27A1 using bioinformatics analysis. Moreover, increased expression of GR was confirmed in the adrenal glands of PDE fetal male offspring. At the cellular level, dexamethasone promotes the nuclear translocation of GR and enables its direct binding to the promoter region of CYP27A1, thereby enhancing transcriptional activation. When an intervention targeting GR is implemented, it effectively counteracts the elevated levels of H3K27ac modification and expression in the CYP27A1 promoter region that are induced by dexamethasone. Moreover, it alleviates the bile acid synthesis resulting from dexamethasone treatment. In conclusion, our findings clearly demonstrate that dexamethasone drives CYP27A1 transcription, accompanied by increased protein expression and bile acid synthesis, all through the activation of GR.

Numerous studies have consistently shown that epigenetic modifications play a pivotal role in regulating the development of multiple organs during the prenatal period and postnatal homeostatic adjustments. As such, they are crucial in the pathogenesis of diseases with a fetal origin.^[^ ^54^, ^55^, ^56^ ^]^ In this study, we focused on the histone acetylation sites in the promoter region of CYP27A1 in the adrenal glands of PDE male offspring. Notably, we observed a consistent elevation in H3K27ac levels in the CYP27A1 promoter region during both the fetal and adult stages. These results suggest that the sustained up – regulation of CYP27A1 expression in the adrenal glands of PDE offspring, both before and after birth, might be linked to the increased H3K27ac levels at its promoter region. Subsequently, we investigated histone acetylation‐related enzymes in fetal adrenal tissue. Our findings revealed that the expression of SETBP1 was significantly reduced in PDE male rats, while the expressions of other enzymes remained largely unchanged. Notably, SETBP1 is a nuclear protein identified through its interaction with the cancer‐associated protein SET.^[^ ^57^ ^]^ It functions as an epigenetic regulatory factor involved in fetal organ development and brain morphogenesis.^[^ ^58^ ^]^ SETBP1 can suppress the transcription of target genes by inducing histone deacetylation.^[^ ^59^ ^]^ To further clarify the regulatory role of SETBP1 on CYP27A1 H3K27ac levels and expression, we treated NCI‐295R cells with dexamethasone. We observed a decrease in SETBP1 expression, accompanied by an increase in CYP27A1 H3K27ac levels and expression. Overexpression of SETBP1 was able to reverse the changes induced by dexamethasone. Moreover, the use of RU486, a GR inhibitor, could counteract the inhibitory effect of dexamethasone on SETBP1. In summary, activation of GR and subsequent suppression of SETBP1 expression by dexamethasone can enhance CYP27A1 H3K27ac levels and expression in adrenal cortex cells. Based on the above‐mentioned findings, the administration of dexamethasone during pregnancy promotes the transcription and expression of CYP27A1 in fetal adrenal glands by activating GR. On one hand, it directly stimulates the transcription and expression of adrenal CYP27A1. On the other hand, it increases the H3K27ac levels and expression of CYP27A1 by inhibiting SETBP1 expression. Ultimately, this leads to an increase in the protein expression of CYP27A1 and enhanced bile acid synthesis.

### 3.4 PDE‐Induced Changes in Bile Acid Metabolism in Adult Offspring Rats and Their Gender‐Specific Mechanisms

In our previous studies, it was observed that PDE exerts differential effects on corticosterone synthesis in adult female and male offspring.^[^ ^13^ ^]^ These findings indicate gender‐specific alterations in adrenal function induced by PDE. In the current study, we concurrently evaluated and compared the alterations in bile acid synthesis and markers of functional damage in the adrenal glands of adult male and female offspring rats. The results showed that in the adrenal glands of PDE male offspring, there was an upregulation of the bile acid synthesis enzyme CYP27A1 expression. This was accompanied by elevated levels of adrenal TBA and reduced blood corticosterone levels. Conversely, in females, opposite trends were observed. Notably, although a low concentration (10 µm) of CDCA has been reported to enhance cortisol synthesis in human adrenal cortex cells,^[^ ^60^ ^]^ our cell‐based experiments demonstrated that a high concentration (200 µm) of CDCA inhibited cortisol synthesis. Overall, these findings underscore the gender differences in PDE‐mediated inhibition or enhancement of corticosterone synthesis in adult offspring rats. Mechanistically, these differences are related to the increased CYP27A1 expression and TBA levels specifically found in males, while the opposite is true for females. In our previous studies, we also noted that developmental toxicity in male offspring is frequently associated with tissue‐specific programming changes of key genes. These changes lead to persistently low‐level functional programming both before and after birth.^[^ ^61^, ^62^ ^]^

In this study, we found a continuous increase in CYP27A1 expression in the adrenal glands of PDE male offspring both prenatally and postnatally. This finding suggests that the elevated CYP27A1 expression and corticosterone synthesis inhibition in adult male offspring originated from the intrauterine environment. Notably, although the CYP27A1 expression was increased in adrenal glands of PDE female fetal rats, it decreased in adult female offspring after birth. Previously, our team has confirmed the gender difference in adult adrenal steroidogenesis function and its intrauterine epigenetic mechanism in the same animal model in this study.^[^ ^13^ ^]^ Specifically, under PDE conditions, in male fetuses, the GR in the adrenal glands is activated, while the androgen receptor (AR) is downregulated. This leads to a reduction in GR‐AR binding, consequently downregulating the levels and expression of H3K14ac in the promoter regions of the transcription factor specificity protein 1 (SP1) and its target gene 11β‐HSD2. Conversely, in female fetuses, the activation of adrenal GR results in an upregulation of the estrogen receptor β (ERβ), increasing GR‐ ERβ binding. As a result, the levels and expression of H3K14ac in the promoter regions of 11β‐HSD2 are upregulated. The 11β‐HSD2 is a metabolic inactivation enzyme for endogenous glucocorticoids (such as corticosterone), and the gender‐differentiated epigenetic modifications and expressions of 11β‐HSD2 in adult male and female offspring can persist after birth. This ultimately gives rise to gender‐specific differences in adrenal steroidogenesis function and blood corticosterone levels in adulthood. We hypothesize that the observed gender differences are most likely attributed to intrauterine dexamethasone exposure and may share a similar sex hormone receptor‐mediated mechanism as described in the literature. This area is worthy of further in‐ depth and systematic investigation in our future research.

### 3.5 CYP27A1 and Nilvadipine can be Used as Early Intervention Targets and Effective Drugs for Adrenal Dysfunction in PDE Adult Offspring, Respectively

A growing body of literature indicates that early post‐natal intervention can effectively reduce the risk of adult‐onset diseases.^[^ ^63^, ^64^ ^]^ Previous research has shown that oral administration of PF915275, also known as 4‐cyano‐biphenyl‐4‐sulfonic acid (6‐aminopyridin‐2‐yl) amide, to adolescent offspring at PW10 for four weeks remarkably alleviates adrenal cortex hyperfunction and hepatic lipid accumulation caused by prenatal exposure to nonylphenol.^[^ ^65^ ^]^ Nilvadipine, a dihydropyridine calcium antagonist, is commonly used in clinical settings for the prevention and treatment of angina, hypertension, cerebral vasospasm, and ischemic heart disease. Notably, it has been discovered that administering 1 mg kg^−1^ of nilvadipine to mice effectively inhibits the activity of CYP27A1.^[^ ^36^ ^]^ However, currently, there is no existing evidence regarding its therapeutic potential in bile acid metabolism related disorders. This lack of evidence presents an opportunity for further research to explore whether nilvadipine could be a viable treatment option for such disorders. Given its established safety profile in other clinical applications, investigating its role in bile acid metabolism related conditions may open new avenues for therapeutic intervention.

To explore whether CYP27A1 can be targeted for early intervention in adrenal dysfunction resulting from Bile acid increase in PDE male offspring, we orally administered nilvadipine (1.4 mg kg^−1^d) to male offspring rats from PW8 to PW12. We then observed its impacts on adrenal function and underlying mechanisms at the whole‐animal level. Histologically, nilvadipine reversed the reduction in the maximal cross sectional area of the adrenal glands, rectified the disrupted adrenal cortex cell structure, alleviated cell swelling with nuclear condensation, and increased the decreased cell count in PDE adult male offspring. Functionally, nilvadipine effectively counteracted the PDE‐induced adrenal dysfunction. This was manifested by reducing the elevated levels of 27‐HC and TBA in the adrenal glands, and increasing the decreased levels of corticosterone in both the adrenal glands and the blood. At the cellular level, we discovered that nilvadipine reversed the elevated levels of 27‐HC and TBA in adrenal cortex cells and the reduced level of corticosterone that were caused by the overexpression of CYP27A1. In summary, our findings suggest that targeting CYP27A1 may represent an effective strategy for preventing or treating bile acid‐induced adrenal damage in adult offspring with PDE. Nilvadipine emerges as a promising candidate drug for such interventional approaches, potentially offering new therapeutic options for addressing adrenal‐related disorders associated with abnormal bile acid metabolism in this context.

## 4 Conclusion

**Figure 8:**
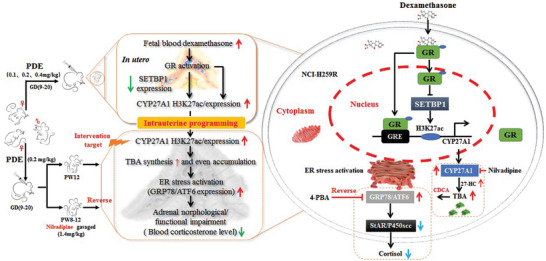
Bile acid increase mechanism of adrenal dysfunction induced by PDE in adult offspring rats and the protective effects of nlivadipine. PDE, prenatal dexamethasone exposure; GR, glucocorticoid receptor; SETBP1, SET binding protein1; CYP27A1, cholesterol 27‐hydroxylase; H3K27ac, histone 3 lysine 27 acetylation; TBA, total bile acids; GRP78, glucose regulated protein 78; ATF6, activating transcription factor 6; CDCA, chenodeoxycholic acid; 4‐PBA, 4‐phenyl butyric acid; 27‐HC, 27‐hydroxycholesterol; StAR, steroidogenic acute regulatory protein; P450scc, cytochrome P450 cholesterol side chain cleavage lyase; GD, gestational day; PW, postnatal week.

PDE can induce dysfunction in adrenal steroid synthesis in adult male offspring. The mechanism is associated with the upregulation of fetal adrenal CYP27A1 promoter region H3K27ac levels and expression mediated by PDE through the GR/SETBP1 signaling pathway. The elevated levels of CYP27A1 H3K27ac and expression may persist postnatally, resulting in local bile acids increase in the adrenal gland, enhanced ERS, and subsequent cellular functional impairment (**Figure** **8**). The above‐mentioned changes are opposite in PDE female offspring. This study systematically elucidates the intrauterine programming mechanism of adrenal function injury caused by local bile acid increase in PDE male adult offspring, proposing CYP27A1 as an early prevention target and nilvadipine as an effective prevention and treatment drug after birth. These findings provide a theoretical basis and early prevention target for the clinical application of dexamethasone‐induced adrenal developmental toxicity in offspring and susceptibility to adult‐related diseases.

## 5 Experimental Section

### Chemicals and Reagents

Dexamethasone was obtained from Shuanghe Pharmaceutical Company (Wuhan, China). Isoflurane was purchased from Baxter Healthcare Co. (Deerfield, IL, USA). Rat corticosterone enzyme‐linked immunosorbent assay (ELISA) kit (61K080) and human cortisol ELISA kit (RE52061) were purchased from Assaypro LLC. (Saint Charles, Missouri, USA) and ILB International GmbH (Hamburg, Germany), respectively. The DMEM and fetal bovine serum (FBS) were provided by Gibco Australia. Penicillin and streptomycin were purchased from Invitrogen (Carlsbad, CA, USA). The overexpression plasmid pcDNA‐SETBP1 (Y8683), pcDNA‐CYP27A1 (Y8712), and the CYP27A1 siRNA plasmid were obtained from Suzhou Gene Pharma Co., Ltd. (Suzhou, China). The TBA kit(E003‐2‐1), nilvadipine (N129505), 4‐PBA (R013902), and CDCA (A012A) were purchased from Guangzhou Ribo Bio Co., Ltd. (Guangzhou, China). Proteinase K (ST533) and mifepristone (RU486) (HY‐13683) were acquired from MedChemExpress Biotechnology Inc (New Jersey, NJ, USA). Total RNA reverse transcription and real‐time quantitative polymerase‐chain‐reaction (RT‐qPCR) kits were procured from Takara Biotechnology Co., Ltd. (Dalian, China). Antibodies for H3K9ac (A7255), H3K14ac (A7254), and H3K27ac (A7253) were purchased from ABclonal Biotech Co., Ltd (Wuhan, China). Immunoglobulin G (IgG) antibody (ab172730) was obtained from Abcam Technology Co., Ltd (Cambridge, UK). Other chemicals and biological reagents were of analytical grade.

### Animals and Treatment

The specific pathogen‐free Wistar rats (No. 2018–2020, license number: SCXK (Hubei), certification number: 42000600002258) with a weight of 278 ± 22 g for males and 205 ± 15 g for females were procured from the Experimental Center of the Hubei Medical Scientific Academy in Wuhan, China. Animal experiments were conducted at the Center for Animal Experiments of Wuhan University, which was accredited by the Association for Assessment and Accreditation of Laboratory Animal Care International (AAALAC International). The experimental protocol was approved by the Committee on Ethics of Animal Experiments at Wuhan University School of Medicine under permit number: 201719. All animal experimental procedures adhered to the guidelines set forth by the Chinese Animal Welfare Committee. The rat feeding procedure is shown in **Figure** **9**.

The rats were maintained under standard conditions, with a room temperature ranging from 18 to 22 °C, humidity between 40% and 60%, and a 12‐h light‐dark cycle. Animals were allowed free access to water and standard feed (11.85% fat, 65.08% carbohydrate, 23.07% protein, total 3.40 kcal g^−1^; Beijing Keao Xieli Feed Co.,Ltd., Beijing, China). After one week of acclimatization, male and female rats were mated at a ratio of 1:2 at 6:00 pm. The presence of sperm in the morning vaginal smear was examined the following day and recorded as GD 0. From GD9 to GD20, pregnant rats in the experimental group (*n *= 12–24) received dexamethasone at doses of 0.1, 0.2, and 0.4 mg kg^−1^·d respectively, while pregnant rats in the control group (*n *= 24) received an equivalent volume of physiological saline solution. On GD20, some pregnant rats were euthanized using anesthesia with isoflurane (2%). Pregnant rats with litter sizes ranging from 8 to 14 were considered eligible for further analysis. Fetal blood samples were collected for serum isolation purposes. Adrenal glands from five fetal rats in different litters per group were selected and fixed overnight in a solution containing paraformaldehyde (4%). This was followed by dehydration in alcohol and embedding in paraffin for subsequent use. The remaining fetal adrenal glands were immediately frozen using liquid nitrogen and stored at ‐80 °C for future analysis.

Another group of pregnant rats (control and 0.2 mg kg^−1^·d dexamethasone) underwent spontaneous delivery at term. On postnatal day (PD) 1, the litter size was randomly adjusted to 8–10 pups per litter to ensure adequate and equal nutrition until weaning. At postnatal week 4 (PW4), ten pups from ten different mothers were randomly selected for each group. The male PDE group received intragastric administration of nilvadipine at a dose of 1.4 mg kg^−1^·d^[^ ^36^ ^]^ from PW8 (infancy) to PW12 (puberty). At PW12, rats were anesthetized and euthanized for collection of adrenal tissues and serum samples. Five adult glands from different litters were selected in each group, fixed overnight in a 4% paraformaldehyde solution, dehydrated in alcohol, and embedded in paraffin for subsequent use. The remaining adrenals were immediately frozen in liquid nitrogen and stored at −80 °C for further analysis.

From GD9‐20, pregnant Wistar rats were subcutaneously injected with dexamethasone 0.1, 0.2, and 0.4 mg kg^−1^·d. At PW8‐12, male rats were administered intragastrically with nilvadipine 1.4 mg kg^−1^·d.

### Histological Examination

The adrenal gland was fixed overnight in a 4% paraformaldehyde solution and subsequently embedded in paraffin. Histologic sections with a thickness of 5 µm were prepared and stained using the hematoxylin‐eosin (H&E) method. Every fifth section from the series was preserved, observed, and photographed under an Olympus AH‐2 light microscope (Olympus, Tokyo, Japan). Cross sectional areas of the cortex were determined through planimetry on these sections, while estimation of cross sectional areas for adrenocortical zones was performed using the Nikon H550S Photo Imaging System (Nikon H550S, Japan).

### Immunohistochemistry Measurements

The immunohistochemical (IHC) procedures were performed using streptavidin‐peroxidase conjugation. Paraffin‐embedded tissues were sectioned, followed by dewaxing with xylene and dehydration with a gradient of ethanol. Tissues were then sealed with sheep serum working fluid and incubated overnight at 4 °C with diluted primary anti‐SETBP1 antibody (1:100 dilution; ABclonal A7212) and anti‐CYP27A1 antibody (1:200 dilution; Abcam ab126785). Phosphate‐buffered saline (PBS) was used as a negative control instead of the primary antibody. Biotin‐labeled secondary antibodies were incubated at 37 °C for 30 min. After counterstaining with hematoxylin, dehydrating, drying, and sealing the tissue sections, at least five random fields from each section were examined using an Olympus AH‐2 Light Microscope (Olympus, Tokyo, Japan). Digital image analysis was performed using Olympus software (Olympus, Tokyo, Japan).

### RNA Sequencing

Total RNA was extracted from adrenal tissues using Trizol according to the manufacturer's protocol. The purity of RNA was assessed by ND‐1000 Nanodrop, and its integrity was evaluated by Agilent 2200 TapeStation (Agilent Technologies, Santa Clara, CA, USA), with each sample having an RIN above 7.0. Briefly, rRNAs were removed from total RNA using EpicentreRibo‐Zero rRNA Removal Kit (illumina, San Diego, California, USA) and fragmented to ≈200 bp. Subsequently, purified RNAs underwent first strand and second strand cDNA synthesis followed by adaptor ligation and enrichment with a low‐cycle as per instructions of NEBNext Ultra RNA Library Prep Kit for Illumina (NEB, Ipswich MA). The purified library products were evaluated using the Agilent 2200 TapeStation and Qubit2.0 before being diluted to 10 pm for cluster generation in situ on the pair‐end flow cell followed by sequencing (2 × 150 bp) HiSeq3000. The clean reads were obtained after removing adapter‐containing reads at low quality or containing poly‐N sequences from raw data. HISAT2 aligned clean reads to the mouse reference genome mm10 with default parameters while HTSeq2.0 (https://pypi.python.org/pypi/HTSeq) converted aligned short reads into read counts for each gene model. Differential expression analysis used DEseq with read counts as input; Benjamini‐Hochberg multiple test correction method enabled differential expression assessment based on criteria of fold change > 2 and adjusted *P*<0.05; all differentially expressed genes were used for heat map analysis.

### Corticosterone and Cortisol Measurement

The concentrations of rat corticosterone and human cellular cortisol were quantified using an ELISA kit in accordance with the manufacturer's protocol. The lower limit of detection for corticosterone was determined to be 0.39 ng mL^−1^. The intra‐assay coefficient of variation (CV) for corticosterone was 5.0%, while the inter‐assay CV was 7.2%. For cortisol, the inter‐assay CV and intra‐assay CV were found to be 5.1% and 9.4%, respectively. Additionally, the cross‐reactivity of the cortisol ELISA assay with related molecules was observed to be less than 0.5%.

### Adrenal and Cellular TBA Concentration Measurement

The cellular TBA concentration was determined using a commercially available kit according to the manufacturer's instructions. Briefly, 3 µL of calibrator and sample were mixed with 200 µL of reagent I and 50 µL of reagent II, followed by thorough mixing and incubation at 37 °C for 3 min. The absorbance was measured at a wavelength of 450 nm and recorded as A1. After an additional incubation period of 2 min, the absorbance was measured again and recorded as A2. The difference in absorbance (△A) was calculated as A2‐A1. For adrenal homogenate samples (5 mg tissue: PBS = 1 mg: 0.9 mL), the same procedure was performed followed by measurement of protein concentration at a wavelength of 570 nm.

Equations:

(1) $$\begin{array}{ccc} \mathrm{TBA}\;\mathrm{concentration}\;\;\mu mol/L & & =\frac{\;\Delta A\;\mathrm{Measured}\;}{\Delta A\;\mathrm{Standard}}\times \\ & & \mathrm{Standard}\;\mathrm{concentration}\;\mu mol/L\;\left(\mathrm{cell}\right) \end{array}$$

(2) $$\begin{array}{ccc} \mathrm{TBA}\;\mathrm{concentration}\; & & \mu mol/L=\frac{\Delta A\;\mathrm{measured}\;}{\Delta A\;\mathrm{Standard}}\times \\ & & \frac{\mathrm{Standard}\;\mathrm{concentration}}{\mathrm{Protein}\;\mathrm{concentration}}\;\mu mol/L\;\left(\mathrm{Tissue}\right) \end{array}$$

### 27‐hydroxycholesterol Concentration Measurement

The concentrations of adrenal and cellular 27‐HC were quantified using an ELISA kit (SU‐B35762, R&D Systems, USA) following the manufacturer's protocol. Briefly, 50 µL of sample, standard, and HRP‐labeled detection antibodies were added to the microwells coated with rat 27‐Hycl antibody. After incubation and washing steps, substrate color development was initiated. The intensity of the resulting color was positively correlated with the amount of 27‐Hycl in the sample. Absorbance (OD value) at a wavelength of 450 nm was measured to determine the its concentration.

### Chromatin Immunoprecipitation (ChIP) Assay

The adrenal tissue of 3 fetal rats were combined from the same litter into one sample, and each group of 6 fetal adrenal samples constituted *n* = 6 per group. The adrenals of adult offspring were not combined due to their sufficient size. In the cell experiment, DNA was extracted by homogenizing all the cells from each plate and was considered a single n value (*n* = 6 per group). The homogenate of adrenal tissues or scraped cells was fixed with 1% formaldehyde for 15 min at 37 °C to cross‐link DNA and its associated proteins, followed by the addition of 125 mm glycine for 8 min to stop the reaction. Cell pellets were then added to 1 mL of Lysis Buffer, and the lysates were sonicated for 4 min (2 s on, 2 s off, output power set at 30%) before being precleared with protein G Sepharose beads (16‐157; Millipore Co., New York, NY, USA). Aliquots of the precleared and sheared chromatin were subsequently immunoprecipitated overnight using antibodies against H3K9ac (1:50 dilution, A7255, ABclonal, Wuhan, China), H3K14ac (1:50 dilution, A7254, ABclonal, Wuhan, China), H3K27ac (1:50 dilution, A7253, ABclonal, Wuhan, China), GR (dilution: 1:1000; CST 1240S, DM, USA) or goat anti‐rabbit IgG (1:50 dilution, AC005, ABclonal, Wuhan, China). The immunoprecipitated DNA‐protein complex with beads was collected by centrifugation and washed sequentially with low‐salt, high‐salt, LiCl immune complex, and Tris‐EDTA washing buffer solutions. Freshly prepared elution buffer (1% SDS, 0.1 m NaHCO3) was used to elute the DNA protein complex. The resulting pellet was resuspended and incubated with proteinase K at a temperature of 65 °C overnight. Finally, purification was performed using a DNA purification kit (639549; Tiangen Biotech Co., Beijing, China) according to the manufacturer's instructions. The purified DNA was then assayed using RT‐qPCR for analysing. The input values were compared to the immunoprecipitated samples, with the IgG negative controls values subtracted as background. The primers are listed in Table S1 (Supporting Information).

### Cell Culture and Treatment

The human adrenocortical cell line (NCI‐H295R) was obtained from Shanghai Zishi Biotechnology Co., Ltd. (Shanghai, China). Cells were cultured in a 5% CO_2_ humidified incubator at 37 °C. The standard medium for NCI‐H295R cells consisted of DMEM supplemented with 5% FBS and 0.1% penicillin/streptomycin. Subsequently, the cells were treated with different concentrations (0, 50, 100, 200, and 400 µm) of CDCA for various durations (0, 12, 24, and 48 h) or different concentrations (0, 20, 100, and 500 nm) of dexamethasone for a duration of 24 h before being harvested for further analysis. To assess the cytotoxicity of dexamethasone and CDCA on NCI‐H295R cells as detected by MTS assay, the absorption intensity was measured at 490 nm using a microplate reader (TECAN, Australia).

Next, NCI‐H295R cells were treated with 200 µm CDCA and 5 mm ERS inhibitor 4‐PBA^[^ ^66^ ^]^ for 24 h to confirm the inhibitory effects of cortisol mediated by activating endoplasmic reticulum. Additionally, NCI‐H295R cells were treated with 500 nm dexamethasone and 2.5 µm GR inhibitor RU486 for 24 h to confirm the inhibitory effect of cortisol mediated by activating GR. Furthermore, NCI‐H295R cells were transfected with either a CYP27A1 siRNA plasmid or a pcDNA3.1(+)‐SETBP1 vector in combination with Lipofectamine 3000 (Invitrogen, Carlsbad, CA, USA). After incubation for 12 h, the cells were treated with dexamethasone at a concentration of 500 nm. To simulate high expression levels of CYP27A1 after birth, NCI‐H295R cells were transfected with a pcDNA3.1 (+)‐CYP27A1 vector in combination with Lipofectamine 3000 and then treated with nilvadipine at a concentration of 5 µm.

### Total RNA Extract and RT‐qPCR Assay

The adrenal gland tissue and NCI‐H295R cells were homogenized using Trizol reagent, followed by extraction of total RNA according to the manufacturer's protocol. The concentration and purity of the isolated total RNA were determined using a spectrophotometer (Nano Drop 2000C, Thermo). Subsequently, the total RNA concentration was adjusted to 1000 ng µL^−1^. Conversion of total RNA to cDNA was performed following the protocol of a reverse transcription kit for total RNA. Primers used in this study are provided in Table S2 (Supporting Information). RT‐qPCR analysis was conducted on an ABI Step One RT‐qPCR thermal cycler (ABI Stepone, NY, USA) with a reaction mixture volume of 10 µL. The mRNA level of glyceraldehyde‐3‐phosphate dehydrogenase (GAPDH) was quantified and utilized as an internal control.

### Western Blot Assay

The adrenal tissues and NCI‐H295R cells were lysed using the RIPA lysis buffer on ice (Beyotime Biotechnology, Shanghai, China). Protein concentration was measured using the BCA protein assay kit (Beyotime Biotechnology, Shanghai, China). After denaturation by adding the loading buffer and heating, equal amounts of protein (50 µg) from each lysate were resolved on 10% SDS polyacrylamide gels and subsequently transferred onto PVDF membranes (Millipore, MA, USA). The membranes were blocked at room temperature for 2 h with 5% skim milk in Tris‐buffered saline containing 1% Tween‐20 (TBST). Subsequently, the membranes were incubated overnight at 4 °C with primary antibodies against GR (dilution: 1:1000; CST 1240S, DM, USA), histone 3 (H3) (dilution: 1:5000; ABclonal A2348), CYP27A1 (dilution: 1:1000; Abcam ab126785), GAPDH (dilution: 1:5000; Abcam ab8245), SETBP1 (dilution: 1:1000; ABclonal A7212), GRP78 (dilution: 1:5000; ABclonal A02411), ATF6 (dilution:1:1000, Santa Cruz sc‐166659), HRP Goat Anti‐Rabbit IgG(dilution: 1:10000; ABclonal AS014) and HRP Goat Anti‐Mouse IgG(dilution: 1:10000; ABclonal AS003). On the following morning, the membranes were washed three times with TBST, and then incubated with secondary antibodies diluted to a ratio of for 2 h on an orbital shaker. After washing the membranes three times again with TBST, the bands were visualized using an ECL kit (Bridgen Beijing China). Their optical densities were measured using a Photo Documentation and Imaging System (BIO‐ID VL Conn France). Densitometry and quantification of the protein bands were performed using Image J Software.

### Dual Luciferase Assay

The dual luciferase assay was performed in accordance with the manufacturer's instructions. NCI‐H295R cells were co‐transfected with 50 ng of CYP27A1 fluorescent plasmid and blank control (NC) using lipofectamine 3000. Lysates were collected at 24 h post‐transfection and treated with dexamethasone (500 nm), RU486 (2.5 µm), and nilvadipine (5 µm). Luciferase activity was quantified using the Dual Luciferase Reporter Assay System (Promega). The Renilla luciferase activity was normalized to the firefly luciferase activity.

### Data and Statistical Analysis

SPSS 26 (SPSS Science Inc., Chicago, Illinois) and Prism 8.0 (GraphPad Software, La Jolla, CA, USA) was employed for statistical analysis and data visualization. All data were obtained from at least three independent experiments and expressed as Mean ± S.E.M. values. RT‐qPCR assay data were normalized to the control group. Statistical analysis was performed using the Two‐tailed Student's t‐test between two groups or one‐way ANOVA among three or more groups, respectively. Sample size (n) for each statistical analysis were depicted in the figure legends.

## Conflict of Interest

The authors declare no conflict of interest.

## Author Contributions

J.C. and W.H. contributed equally to this work. J.C. performed conceptualization and wrote the original draft; W.H. performed reviewing, revising, and editing; Y.C. performed methodology; A.A. performed visualization; Z.Z. and L.R. performed formal analysis; H.Y. performed wrote, reviewed, and edited the draft; H.W. performed project administration, funding acquisition, and wrote, reviewed, and edited the draft. The manuscript has been reviewed and approved by all authors.

## Supporting information



Supporting Information

## Data Availability

Research data are not shared.

## References

[1] A. Walters , C. McK inlay , P. Middleton , J. E. Harding , C. A. Crowther , Cochrane Database Syst. Rev. 2022, 4, CD003935. 35377461 10.1002/14651858.CD003935.pub5 PMC8978608

[2] M. K. Auer , A. Nordenstrom , S. Lajic , N. Reisch , Lancet 2023, 401, 227. 36502822 10.1016/S0140-6736(22)01330-7

[3] E. McGoldrick , F. Stewart , R. Parker , S. R. Dalziel , Cochrane Database Syst. Rev. 2020, 12, CD004454. 33368142 10.1002/14651858.CD004454.pub4 PMC8094626

[4] M. Liu , B. Chen , L. Pei , Q. Zhang , Y. Zou , H. Xiao , J. Zhou , L. Chen , H. Wang , Toxicology 2018, 408, 1. 29902490 10.1016/j.tox.2018.06.005

[5] H. Xiao , Y. Wen , Z. Pan , Y. Shangguan , J. Qin , Y. Tan , H. Jiang , B. Li , Q. Zhang , L. Chen , H. Wang , Cell Death Dis. 2018, 9, 638. 29844424 10.1038/s41419-018-0701-z PMC5974192

[6] F. Lv , Y. Wan , Y. Chen , L. Pei , D. Luo , G. Fan , M. Luo , D. Xu , H. Wang , Endocrinology 2018, 159, 1401. 29370380 10.1210/en.2018-00044

[7] H. Liu , B. He , W. Hu , K. Liu , Y. Dai , D. Zhang , H. Wang , Biochem. Pharmacol. 2021, 185, 114420. 33460628 10.1016/j.bcp.2021.114420

[8] L. Li , W. Hu , K. Liu , D. Zhang , M. Liu , X. Li , H. Wang , Toxicol. Appl. Pharmacol. 2020, 395, 114979. 32234517 10.1016/j.taap.2020.114979

[9] S. Hu , Y. Yi , T. Jiang , Z. Jiao , S. Dai , X. Gong , K. Li , H. Wang , D. Xu , Arch. Toxicol. 2020, 94, 3201. 32494933 10.1007/s00204-020-02796-1

[10] Y. Shangguan , Z. Wu , X. Xie , S. Zhou , H. He , H. Xiao , L. Liu , J. Zhu , H. Chen , H. Han , H. Wang , L. Chen , Biochem. Pharmacol. 2021, 185, 114414. 33434537 10.1016/j.bcp.2021.114414

[11] G. Chen , C. Ai , F. Duan , Y. Chen , J. Cao , J. Zhang , Y. Ao , H. Wang , Cell Biol. Toxicol. 2023, 39, 2051. 35246761 10.1007/s10565-021-09691-0

[12] Y. Chen , X. Xia , M. Fang , G. Chen , J. Cao , H. Qu , H. Wang , Sci. Total Environ. 2021, 797, 149084. 34303245 10.1016/j.scitotenv.2021.149084

[13] Y. Chen , D. Xu , X. Xia , G. Chen , H. Xiao , L. Chen , H. Wang , Pharmacol. Res. 2021, 174, 105942. 34656764 10.1016/j.phrs.2021.105942

[14] A. Di Ciaula , G. Garruti , R. Lunardi Baccetto , E. Molina‐Molina , L. Bonfrate , D. Q. Wang , P. Portincasa , Ann. Hepatol. 2017, 16, s4. 29080336 10.5604/01.3001.0010.5493

[15] S. H. Mohammed , M. Mirdamadi , K. F. Szucs , R. Gaspar , Biochem. Pharmacol. 2024, 222, 116063. 38373593 10.1016/j.bcp.2024.116063

[16] F. Song , Y. Chen , L. Chen , H. Li , X. Cheng , W. Wu , JAMA Netw. Open 2021, 4, 2117409. 10.1001/jamanetworkopen.2021.17409 PMC8290304 34279647

[17] L. Liu , S. Zhou , A. Zaufel , Z. Xie , S. Racedo , M. Wagner , G. Zollner , P. Fickert , Q. Zhang , Biochem. Biophys. Res. Commun. 2024, 692, 149342. 38061283 10.1016/j.bbrc.2023.149342

[18] M. McMillin , G. Frampton , M. Quinn , A. Divan , S. Grant , N. Patel , K. Newell‐Rogers , S. DeMorrow , Mol. Endocrinol. 2015, 29, 1720. 26431088 10.1210/me.2015-1087 PMC4664228

[19] A. D. McNeilly , D. P. Macfarlane , E. O'Flaherty , D. E. Livingstone , T. Mitic , K. M. McConnell , S. M. McKenzie , E. Davies , R. M. Reynolds , H. C. Thiesson , O. Skott , B. R. Walker , R. Andrew , J. Hepatol. 2010, 52, 705. 20347173 10.1016/j.jhep.2009.10.037 PMC2877801

[20] R. Taylor , Z. Yang , Z. Henry , G. Capece , V. Meadows , K. Otersen , V. Basaly , A. Bhattacharya , S. Mera , P. Zhou , L. Joseph , I. Yang , A. Brinker , B. Buckley , B. Kong , G. L. Guo , Toxicol. Sci. 2024, 199, 316. 38526215 10.1093/toxsci/kfae029 PMC12104498

[21] W. Jia , M. Wei , C. Rajani , X. Zheng , Protein Cell 2021, 12, 411. 33252713 10.1007/s13238-020-00804-9 PMC8106556

[22] V. Sigurdsson , H. Takei , S. Soboleva , V. Radulovic , R. Galeev , K. Siva , L. M. Leeb‐Lundberg , T. Iida , H. Nittono , K. Miharada , Cell Stem Cell 2016, 18, 522. 26831518 10.1016/j.stem.2016.01.002

[23] O. V. Fedorova , V. I. Zernetkina , V. Y. Shilova , Y. N. Grigorova , O. Juhasz , W. Wei , C. A. Marshall , E. G. Lakatta , A. Y. Bagrov , Circ. Cardiovasc. Genet. 2015, 8, 736. 26374826 10.1161/CIRCGENETICS.115.001217 PMC4618091

[24] Y. Liu , M. A. K. Azad , W. Zhang , L. Xiong , F. Blachier , Z. Yu , X. Kong , J. Anim. Sci. Biotechnol. 2022, 13, 117. 36320049 10.1186/s40104-022-00772-6 PMC9628178

[25] S. Majait , E. C. E. Meessen , F. M. Vaz , E. M. Kemper , S. V. Nierop , S. W. Olde Damink , F. G. Schaap , J. A. Romijn , M. Nieuwdorp , A. Verrips , F. K. Knop , M. R. Soeters , Nutrients 2023, 15, 4625. 37960277 10.3390/nu15214625 PMC10648145

[26] G. Chen , H. Xiao , J. Zhang , H. Zhang , B. Li , T. Jiang , Y. Wen , Y. Jiang , K. Fu , D. Xu , Y. Guo , Y. Ao , H. Bi , H. Wang , Toxicol. Lett. 2019, 316, 136. 31520701 10.1016/j.toxlet.2019.09.007

[27] R. Barouki , E. Melen , Z. Herceg , J. Beckers , J. Chen , M. Karagas , A. Puga , Y. Xia , L. Chadwick , W. Yan , K. Audouze , R. Slama , J. Heindel , P. Grandjean , T. Kawamoto , K. Nohara , Environ. Int. 2018, 114, 77. 29499450 10.1016/j.envint.2018.02.014 PMC5899930

[28] L. A. Joss‐Moore , K. H. Albertine , R. H. Lane , Mol. Genet. Metab. 2011, 104, 61. 21835665 10.1016/j.ymgme.2011.07.018 PMC3171512

[29] Q. Xu , T. Wang , Y. Cao , Y. Qi , Y. Cao , C. Fu , X. Tao , T. Yu , W. Lu , X. Jiang , Zhonghua WeiZhongBing JiJiu YiXue 2020, 32, 33. 10.3760/cma.j.cn121430-20190725-00006 32148228

[30] A. M. Shalaby , A. M. Aboregela , M. A. Alabiad , D. F. El Shaer , Microsc. Microanal. 2020, 26, 509. 32366353 10.1017/S1431927620001397

[31] J. Y. Chiang , Compr. Physiol. 2013, 3, 1191. 23897684 10.1002/cphy.c120023 PMC4422175

[32] Y. Shen , W. Wei , D. X. Zhou , Trends Plant Sci. 2015, 20, 614. 26440431 10.1016/j.tplants.2015.07.005

[33] L. Liu , K. Panzitt , S. Racedo , M. Wagner , W. Platzer , A. Zaufel , V. Theiler‐Schwetz , B. Obermayer‐Pietsch , H. Mueller , G. Hoefler , A. Heinemann , G. Zollner , P. Fickert , Liver Int. 2019, 39, 2112. 30664326 10.1111/liv.14052 PMC6899711

[34] W. Zeng , Y. H. Guo , W. Qi , J. G. Chen , L. L. Yang , Z. F. Luo , J. Mu , B. Feng , Life Sci. 2014, 103, 15. 24650493 10.1016/j.lfs.2014.03.007

[35] C. J. Y. L. J. M. Ferrell , Liver Res. 2020, 4, 47. 34290896 10.1016/j.livres.2020.05.001 PMC8291349

[36] M. Lam , N. Mast , I. A. Pikuleva , Mol. Pharmacol. 2018, 93, 101. 29192124 10.1124/mol.117.110742 PMC5749491

[37] K. Haram , J. H. Mortensen , E. F Magann , J. C. Morrison , J. Matern. Fetal Neonatal. Med. 2017, 30, 1437. 27487405 10.1080/14767058.2016.1219716

[38] S. Reagan‐Shaw , M. Nihal , N. Ahmad , FASEB J. 2008, 22, 659. 17942826 10.1096/fj.07-9574LSF

[39] R. M. Paragliola , G. Papi , A. Pontecorvi , S. M. Corsello , Int. J. Mol. Sci. 2017, 18, 2201. 29053578 10.3390/ijms18102201 PMC5666882

[40] T. A. Quinn , U. Ratnayake , M. Castillo‐Melendez , K. M. Moritz , H. Dickinson , D. W. Walker , J. Endocrinol. 2014, 221, 347. 24594617 10.1530/JOE-13-0514

[41] K. N. Shivaram , B. M. Winklhofer‐Roob , M. S. Straka , M. W. Devereaux , G. Everson , G. W. Mierau , R. J. Sokol , Free Radical Biol. Med. 1998, 25, 480. 9741584 10.1016/s0891-5849(98)00077-x

[42] R. Sharma , F. Majer , V. K. Peta , J. Wang , R. Keaveney , D. Kelleher , A. Long , J. F. Gilmer , Bioorg. Med. Chem. 2010, 18, 6886. 20713311 10.1016/j.bmc.2010.07.030

[43] C. Wang , X. Chen , S. F. Zhou , X. Li , Med. Sci. Monit. 2011, 17, CR265. 21525808 10.12659/MSM.881766 PMC3539589

[44] C. Amiel‐Tison , D. Cabrol , R. Denver , P. H. Jarreau , E. Papiernik , P. V. Piazza , Early Hum. Dev. 2004, 78, 81. 15223113 10.1016/j.earlhumdev.2004.03.007

[45] B. L. Copple , H. Jaeschke , C. D. Klaassen , Semin. Liver Dis. 2010, 30, 195. 20422501 10.1055/s-0030-1253228

[46] E. Santos Silva , S. Rocha , R. Candeias Ramos , H. Coutinho , C. Catarino , F. Teixeira , G. Henriques , A. I. Lopes , A. Santos‐Silva , D. Brites , Pediatr. Res. 2023, 93, 1856. 36272998 10.1038/s41390-022-02350-y

[47] K. Kwon , Y. S Kwon , S. W. Kim , K. Yu , K. H. Lee , O. Y. Kwon , Mol. Med. Rep. 2017, 16, 380. 28498401 10.3892/mmr.2017.6582

[48] L. Wu , X. Huang , Y. Kuang , Z. Xing , X. Deng , Z. Luo , Drug Des. Dev. Ther. 2019, 13, 2787. 10.2147/DDDT.S209947 PMC6697672 31496655

[49] W. S. Choi , G. Lee , W. H. Song , J. T. Koh , J. Yang , J. S. Kwak , H. E. Kim , S. K. Kim , Y. O. Son , H. Nam , I. Jin , Z. Y. Park , J. Kim , I. Y. Park , J. I. Hong , H. A. Kim , C. H. Chun , J. H. Ryu , J. S. Chun , Nature 2019, 566, 254. 30728500 10.1038/s41586-019-0920-1

[50] D. W. Russell , Annu. Rev. Biochem. 2003, 72, 137. 12543708 10.1146/annurev.biochem.72.121801.161712

[51] S. C. Biddie , B. L. Conway‐Campbell , S. L. Lightman , Rheumatology (Oxford) 2012, 51, 403. 21891790 10.1093/rheumatology/ker215 PMC3281495

[52] M. V. G. S. G. Matthews , Nat. Rev. Endocrinol. 2014, 10, 403. 24863383 10.1038/nrendo.2014.74

[53] W. Tang , M. Norlin , K. Wikvall , Biochim. Biophys. Acta 2008, 1781, 718. 18817892 10.1016/j.bbalip.2008.08.005

[54] R. H. Lane , Clin. Perinatol. 2014, 41, 815. 25459776 10.1016/j.clp.2014.08.006

[55] M. A. Charles , C. Delpierre , B. Breant , Med. Sci. 2016, 32, 15. 10.1051/medsci/20163201004 26850602

[56] K. M. Godfrey , P. M. Costello , K. A. Lillycrop , Epigenet. Metab. Programm. 2016, 85, 71.

[57] J. H. Whitlock , E. J. Wilk , T. C. Howton , A. D. Clark , B. N. Lasseigne , PLoS One 2024, 19, 0296328. 10.1371/journal.pone.0296328 PMC10760659 38165902

[58] R. Piazza , V. Magistroni , S. Redaelli , M. Mauri , L. Massimino , A. Sessa , M. Peronaci , M. Lalowski , R. Soliymani , C. Mezzatesta , A. Pirola , F. Banfi , A. Rubio , D. Rea , F. Stagno , E. Usala , B. Martino , L. Campiotti , M. Merli , F. Passamonti , F. Onida , A. Morotti , F. Pavesi , M. Bregni , V. Broccoli , M. Baumann , C. Gambacorti‐Passerini , Nat. Commun. 2018, 9, 2192. 29875417 10.1038/s41467-018-04462-8 PMC5989213

[59] B. A. Vishwakarma , N. Nguyen , H. Makishima , N. Hosono , K. O Gudmundsson , V. Negi , K. Oakley , Y. Han , B. Przychodzen , J. P. Maciejewski , Y. Du , Leukemia 2016, 30, 200. 26205084 10.1038/leu.2015.200 PMC4703539

[60] L. Liu , K. Panzitt , S. Racedo , M. Wagner , W. Platzer , A. Zaufel , V. Theiler‐Schwetz , B. Obermayer‐Pietsch , H. Muller , G. Hofler , A. Heinemann , G. Zollner , P. Fickert , Liver Int. 2019, 39, 2112. 30664326 10.1111/liv.14052 PMC6899711

[61] J. Lu , Q. Li , G. Ma , C. Hong , W. Zhang , Y. Wang , H. Wang , Food Chem. Toxicol. 2020, 141, 111419. 32437893 10.1016/j.fct.2020.111419

[62] M. Liu , Q. Zhang , L. Pei , Y. Zou , G. Chen , H. Wang , Epigenetics 2019, 14, 245. 30821590 10.1080/15592294.2019.1581595 PMC6557552

[63] A. Ahmadalipour , S. Ghodrati‐Jaldbakhan , S. A. Samaei , A. Rashidy‐Pour , Neurobiol. Learn. Memory 2018, 147, 54. 10.1016/j.nlm.2017.11.013 29175674

[64] Y.‐J. Lin , I. C. Lin , H.‐R. Yu , J.‐M. Sheen , L.‐T. Huang , Y.‐L. Tain , Oxid. Med. Cell. longevity 2018, 2018, 5343462. 10.1155/2018/5343462 PMC5832129 29636848

[65] L. L. Chang , W. A. Wun , P. S. Wang , BMC Pharmacol. Toxicol. 2018, 19, 45. 30021644 10.1186/s40360-018-0235-0 PMC6052566

[66] X. Shi , L. Gong , Y. Liu , K. Hou , Y. Fan , C. Li , T. Wen , X. Qu , X. Che , Epigenetics 2020, 15, 632. 31814524 10.1080/15592294.2019.1700032 PMC7574398

