# Supplementary material for: Adrenal High‐Expressional CYP27A1 Mediates Bile Acid Increase and Functional Impairment in Adult Male Offspring by Prenatal Dexamethasone Exposure
Source: Adv Sci (Weinh). 2025 Feb 14;12(14):2413299. doi: 10.1002/advs.202413299 (PMC11984885; doi:10.1002/advs.202413299)
Supplement: Supplementary file 1 — Supporting Information [file ADVS-12-2413299-s001.docx]

***Supporting Information***

**Adrenal High-Expressional CYP27A1 Mediates Bile Acid Increase and Functional Impairment in Adult Male Offspring by Prenatal Dexamethasone Exposure**

***Corresponding author:**

Hui Wang, PhD.,

School of Basic Medical Sciences, Wuhan University, Wuhan 430071, China

E-mail: wanghui19@whu.edu.cn.

**Supplementary Tables：**

**Table S1. CYP27A1 oligonucleotide primers and PCR conditions for ChIP-PCR**.

| Antibodies | Species | Forward primer | Reverse primer |
| --- | --- | --- | --- |
| H3K9ac  H3K14ac  H3K27ac | Rat | CCTTTAAAGTCGCCAGGTCG | TTAGAGCCAGAGA CTACGCG |
|  | Human | AGTGCATGGCACAATCTCAG | CAGCACCCAACCTAGACCAT |
| GR | Human | CAGAGTTCAGACCAAGCGA | GCGGTGACTCGGGGATATC |

H3K9ac, histone 3 Lysine 9 acetylation; H3K14ac, histone 3 Lysine 14 acetylation; H3K27ac, histone 3 lysine 27 acetylation; GR, glucocorticoid receptor.

**Table S2.** Oligonucleotide primers and PCR conditions for real-time quantitative PCR.

| Species | Genes | Forward primer | Reverse primer | Annealing (°C) |
| --- | --- | --- | --- | --- |
| Rat | GAPDH | AGTTAATGCCGCCCCTTACC | CAGGGCTGACTACAAACCCA | 63 (30 s) |
|  | GR | CACCCATGACCCTGTCAGTC | AAAGCCTCCCTCTGCTAACC | 63 (30 s) |
|  | SETBP1 | CCAGTACGGCAAGGAAGA | GAGGCTGAAGAGGATGAGGA | 63 (30 s) |
|  | CYP27A1 | CTTTGGCTATGGGGTTCGGT | CACAGCCCTCCACACATTCT | 60 (30 s) |
|  | GRP78 | GTGCCCACCAAGAAGTCTCA | ACACTGGTCCAACTAAGCACC | 60 (30 s) |
|  | ATF6 | GCATTCCGGGTGGAAGCTAT | TCCTGCCCACATTTTGGTGT | 60 (30 s) |
|  | StAR | GGGAGATGCCTGAGCAAAGC | GCTGGCGAACTCTATCTGGGT | 60 (30 s) |
|  | P450scc | GCTGCCTGGGATGTGATTTTC | GATGTTGGCCTGGATGTTCTTG | 60 (30 s) |
|  | BSEP | AACTCCGTAGTGGCTGCTCA | TAACCGTCCCCTGCTTTGTT | 58 (30 s) |
|  | MRP1 | ATAAAGCGGGTTCCTGCCTG | CTCAACCTTCGGTTTGCAGC | 60 (30 s) |
|  | MRP3 | AGAAGGCAGGTGTTTCCAGC | CACTAGTTGCAGCGAGGTCA | 60 (30 s) |
|  | NTCP | TGGACTTGAGGACGATCCCT | ACGTTTACCGCCCAAGAGAG | 60 (30 s) |
| Human | GAPDH | GCAACTAGGATGGTGTGGCT | TCCCATTCCCCAGCTCTCATA | 60 (30 s) |
|  | GR | AAGGTCTGCGCTACACAGTT | TGCCTTTCCCTGGAATTCTG | 60 (30 s) |
|  | SETBP1 | ACAAGAAAGGGGGCAAGTGA | AGAGCCATCTCCAAGTCCCAA | 60 (30 s) |
|  | CYP27A1 | TCTGTGCCCTTTGGCTATGG | TGTGGTTGGGTGGATTGTGT | 60 (30 s) |
|  | GRP78 | TCAAAGACCGTGTTCTCGGG | TCTGTCGGGTTTTTCTGCCA | 60 (30 s) |
|  | ATF6 | TGGTGTCCAATCCCTTGGTG | GATGTGCAATTCAGCACACTCT | 60 (30 s) |
|  | StAR | TCTCGAGCCTGAGAGGATGAA | TCAGGGCAAAAGTGCAGACA | 60 (30 s) |
|  | P450scc | TACTGAGGGTCTAGGAAGTGGT | ACCAGGAGGGACAGTACGTT | 60 (30 s) |

GR, glucocorticoid receptor; SETBP1, SET binding protein1; CYP27A1, cholesterol 27-hydroxylase; GRP78, glucose regulated protein 78; ATF6, activating transcription factor 6; StAR, steroidogenic acute regulatory protein; P450scc, cytochrome P450 cholesterol side chain cleavage enzyme; NTCP, sodium taurocholate cotransporting polypeptide; MRP1/3 cholesterol efflux gene multidrug resistance-associated protein1/3; BSEP, bile salt export pump; GAPDH, glyceraldehyde 3-phosphate dehydrogenase.

**Supplementary Figures**

**
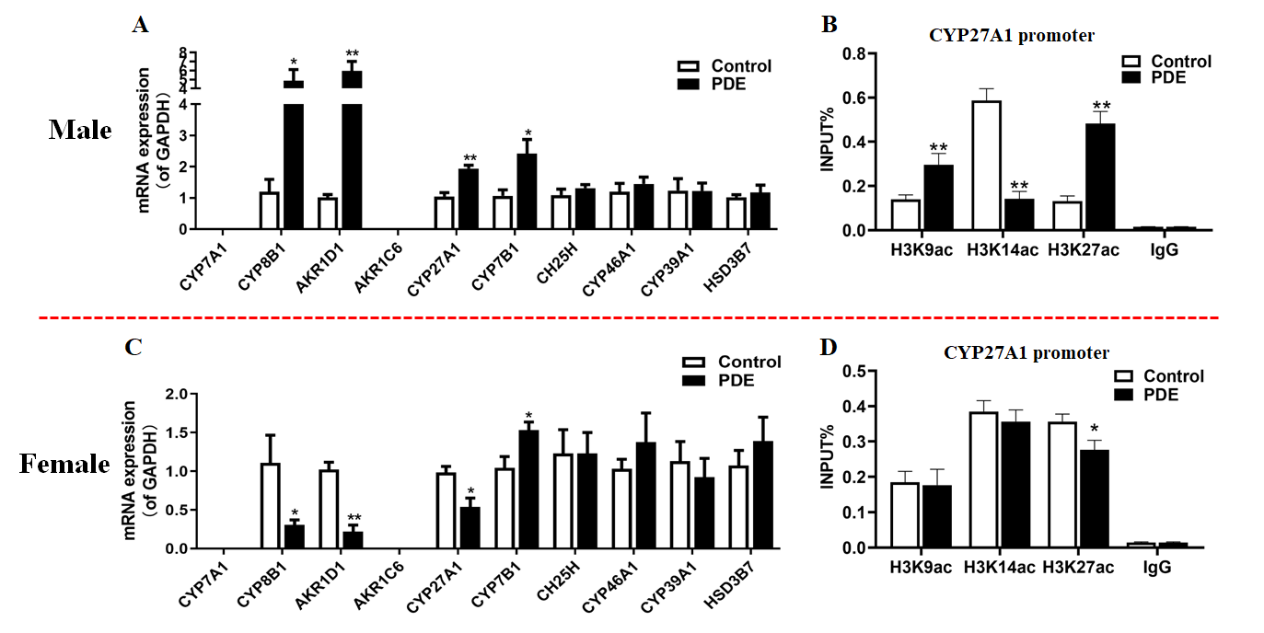
**

**Figure S1. Effects of PDE on adrenal bile acid synthases and histone acetylation of CYP27A1 in male and female adult offspring rats.** A, C: The male and female mRNA expression of bile acid synthases-related enzymes; B, D: The male and female CYP27A1 histone acetylation levels. Data are shown as the mean ± S.E.M., *n*=6 for RT-qPCR and *n*=3 for ChIP assay. *^*^P*<0.05, *^**^P*<0.01 *vs.* control. PDE, CYP7A1, cholesterol 7α-hydroxylase1; prenatal dexamethasone exposure; CYP8B1, cytochrome P450 family 8 subfamily B member 1; CYP27A1, cytochrome P450 family 27 subfamily A member 1; CYP7B1, oxysterol 7α-hydroxylase; AKR1D1, aldo-keto reductase family 1 member D1; AKR1C6, aldo-keto reductase family 1 member C6; CYP7B1, cytochrome P450 family 7 subfamily B member 1; CH25H, cholesterol 25-hydroxylase; CYP46A1, cytochrome P450 family 46 subfamily A member 1; CYP39A1, cytochrome P450 family 39 subfamily A member 1; HSD3B7, 3 beta-hydroxysteroid dehydrogenase type 7; H3K9ac, histone 3 lysine 9 acetylation; H3K14ac, histone 3 lysine 14 acetylation; H3K27ac, histone 3 lysine 27 acetylation; GAPDH, glyceraldehyde 3-phosphate dehydrogenase.


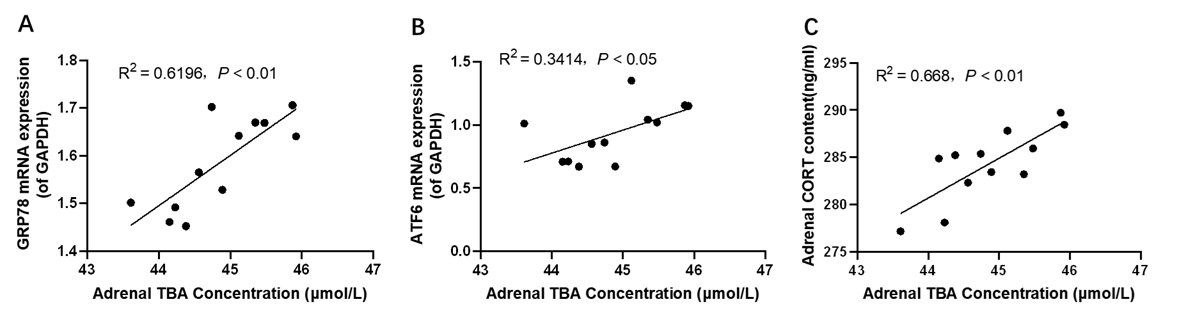


**Figure S2.** **Correlation analysis of the concentration of total bile acids in the adrenal glands of adult male offspring of PDE with endoplasmic reticulum stress markers (GRP78 and ATF6) and corticosterone concentration.** A, B: Correlation between the concentration of total bile acids and the mRNA of GRP78 and ATF6; C: Correlation between the concentration of total bile acids and corticosterone content. Data are shown as the mean ± S.E.M., *n*=10. *^*^P*<0.05, *^**^P*<0.01 *vs.* respective control. GRP78, glucose regulated protein 78; ATF6, activating transcription factor 6; CORT, corticosterone.


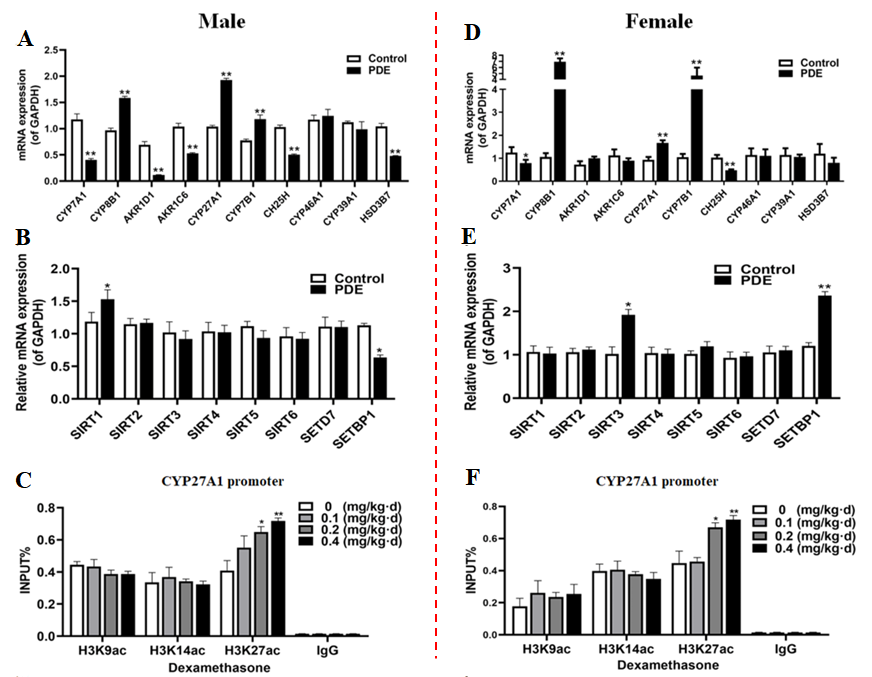


**Figure S3. Effects of PDE on the expression of adrenal bile acid synthetases and histone deacetylases, and histone acetylation of CYP27A1 in the male and female fetal adrenal glands.** A, D: The male and female mRNA expression of bile acid synthetases; B, E: The male and female CYP27A1 histone acetylation levels; C, F: The male and female SIRT 1-6, SETD7 and SETBP1 mRNA expression. Data are shown as the mean ± S.E.M., *n*=6 for RT-qPCR(six pairs of fetal adrenals from two littermates were pooled for homogenization into one sample) and *n*=3 for ChIP assay. *^*^P*<0.05, *^**^P*<0.01 *vs.* control. PDE, prenatal dexamethasone exposure; CYP7A1, cholesterol 7-alpha-hydroxylase1; CYP8B1, cytochrome P450 family 8 subfamily B member 1; CYP27A1, cytochrome P450 family 27 subfamily A member 1; CYP7B1, oxysterol 7α-hydroxylase; AKR1D1, aldo-keto reductase family 1 member D1; AKR1C6, aldo-keto reductase family 1 member C6; CYP7B1, cytochrome P450 family 7 subfamily B member 1; CH25H, cholesterol 25-hydroxylase; CYP46A1, cytochrome P450 family 46 subfamily A member 1; CYP39A1, cytochrome P450 family 39 subfamily A member 1; HSD3B7, 3 beta-hydroxysteroid dehydrogenase type 7; H3K9ac, histone 3 Lysine 9 acetylation; H3K14ac, histone 3 Lysine 14 acetylation; H3K27ac, histone 3 lysine 27 acetylation; SIRT, Sirtuin; SETD7, SET domain containing 7; GAPDH, glyceraldehyde 3-phosphate dehydrogenase.


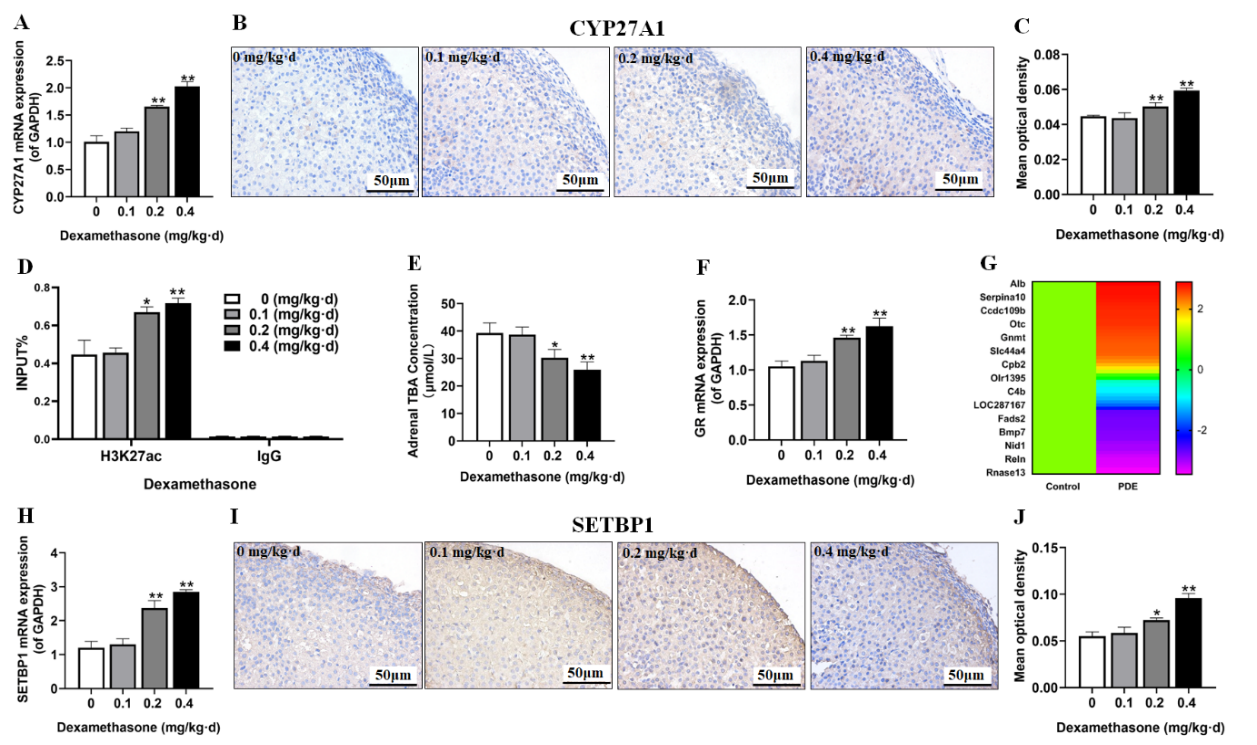


**Figure S4. Effects of PDE on adrenal GR/SETBP1/CYP27A1 expression and TBA level in the female fetal rats at gestational day 20.** A: The CYP27A1 mRNA expression; B, C: The CYP27A1 protein expression by IHC staining (400×); D: The CYP27A1 H3K27ac level; E:The TBA level; F:The GR mRNA expression; G: Heat map of differentially expressed genes;H: The SETBP1 mRNA expression; I, J: The SETBP1 protein expression by IHC staining (400×). six pairs of fetal adrenals from two littermates were pooled for homogenization into one sample. Data are shown as the mean ± S.E.M., *n*=5 for IHC staining(sections of each group were selected and five random fields of each section scored), *n*=3 for ChIP assay and *n*=8 for others. *P* value was calculated by independent samples *t*-test. *^*^P*<0.05, *^**^P*<0.01 *vs.* control. PDE, prenatal dexamethasone exposure; G; GR, glucocorticoid receptor; SETBP1, SET binding protein1; CYP27A1, cytochrome P450 family 27 subfamily A member 1; TBA, total bile acid; H3K27ac, histone 3 lysine 27 acetylation; IgG, immunoglobulin GAPDH, glyceraldehyde 3-phosphate dehydrogenase; IHC, immuohistochemical.
